# Plants, food and treatments used by BaKongo tribes in Uíge (northern Angola) to affect the quality and quantity of human breast milk

**DOI:** 10.1186/s13006-020-00329-1

**Published:** 2020-10-23

**Authors:** Gesine Jendras, Mawunu Monizi, Christoph Neinhuis, Thea Lautenschläger

**Affiliations:** 1 Department of Biology, Institute of Botany, Faculty of Science, Technische Universität Dresden, 01062 Dresden, Germany; 2 University of Kimpa Vita, Province of Uíge, Rua Henrique Freitas No. 1, Bairro Popular, Uíge, Angola

**Keywords:** Breastfeeding, Galactagogue, Infant mortality, Traditional knowledge, BaKongo people, Angola

## Abstract

**Background:**

Angola has one of the highest annual under-five mortality rates in in the world and malnutrition poses a severe problem in the country. This study is the first to focus on the traditional knowledge of plants, foods, and treatments used by the local population in the province of Uíge to affect the quality and quantity of human breast milk, since decades of independence and civil war impeded ethnobotanical studies in this area.

**Methods:**

This study was conducted in eight municipalities in the province of Uíge, Northern Angola in February and March 2018. In 265 semi-structured interviews, 360 informants in 40 rural villages were asked about plants, food, and treatments used to affect the quality and quantity of human breast milk. Additionally, information on child mortality and the duration of breastfeeding were collected. Whenever possible, plant specimens were collected for later identification. To determine the local importance of the collected plants, food, and treatments, the Relative Frequency of Citations was calculated.

**Results:**

Most women reported to have no problems with their breast milk production. The duration of breastfeeding meets the recommendations of the World Health Organization (WHO). Across all use categories, 69 plants from 36 plant families, and 21 other foods and treatments could be identified.

**Conclusions:**

The study shows an overview of a variety of plants, foods, and treatments used by mothers as galactagogues, to “clean” or to reduce their breast milk and those which they avoided to use during the lactation period. There is great potential for further research into this traditional knowledge. Also, further analysis of some of the plants could be of interest.

## Background

Angola still suffers from one of the highest under-five mortality rates in the world, even though the numbers have decreased in recent years [1] due to different reasons. However, these data should be regarded with care because Angola failed to collect data on deaths occurring at home or at small rural health stations [2] so that no reliable information on mortality is available. According to Rosário [2], the major causes of death of Angolan children are malaria and intestinal infections followed by malnutrition and acute respiratory infections. No data about child malnutrition for the province of Uíge in northern Angola are available, but investigations conducted by UNICEF show a large problem with malnutrition in other regions of Angola and in the Democratic Republic of Congo [3, 4]. According to a study conducted in the neighbouring province of Bengo, malnutrition is the third-most cause of death in one to 4 year old children [2]. Malnutrition can promote the outbreak of gastrointestinal infections like diarrhoea [5, 6] and is a supporting factor in acute respiratory infections and malaria [6] as well. This could indicate that many child deaths are related to malnutrition and that a sufficient supply with nutrients is the main prerequisite to ensure the well-being of children.

In the fight against malnutrition, a prolonged lactation period is recommended by WHO [7, 8]. The nutritional status of the mother is crucial for the quantity and quality of breast milk. Essential micronutrients provided by the mother through her milk can be categorised into two groups. Group 1 nutrients include thiamine, riboflavin, vitamin B6, vitamin B12, choline, retinol, vitamin A, vitamin D, selenium and iodine. Their amount in the breast milk depends on the mother’s diet. Group 2 nutrients are those, which are constantly secreted in the mother’s milk. This group includes nutrients like folate, calcium, iron, copper and zinc the content of which does not depend on the mother’s nutrition. Nevertheless, the uptake of these nutrients from food is beneficial for the mother as well. An adequate supply especially of Group 1 nutrients is recommended for nursing mothers to ensure a good health status of both the infant and the mother [9].

Several plants are known to influence the quantity and quality of breast milk. According to Neuwinger [10], these galactagogues are agents that promote the secretion of milk or increase milk flow. The use of galactagogue herbs by women around the world have been recorded in previous studies [11–15]. A study conducted in the neighbouring country also showed the use of five galactagogues by the rural population [16].

With this study, we provide a first overview of traditional plants, food and treatments used by BaKongo tribes in Uíge affecting the quality and quantity of human breast milk.

## Methods

### Study area

**Fig. 1:**
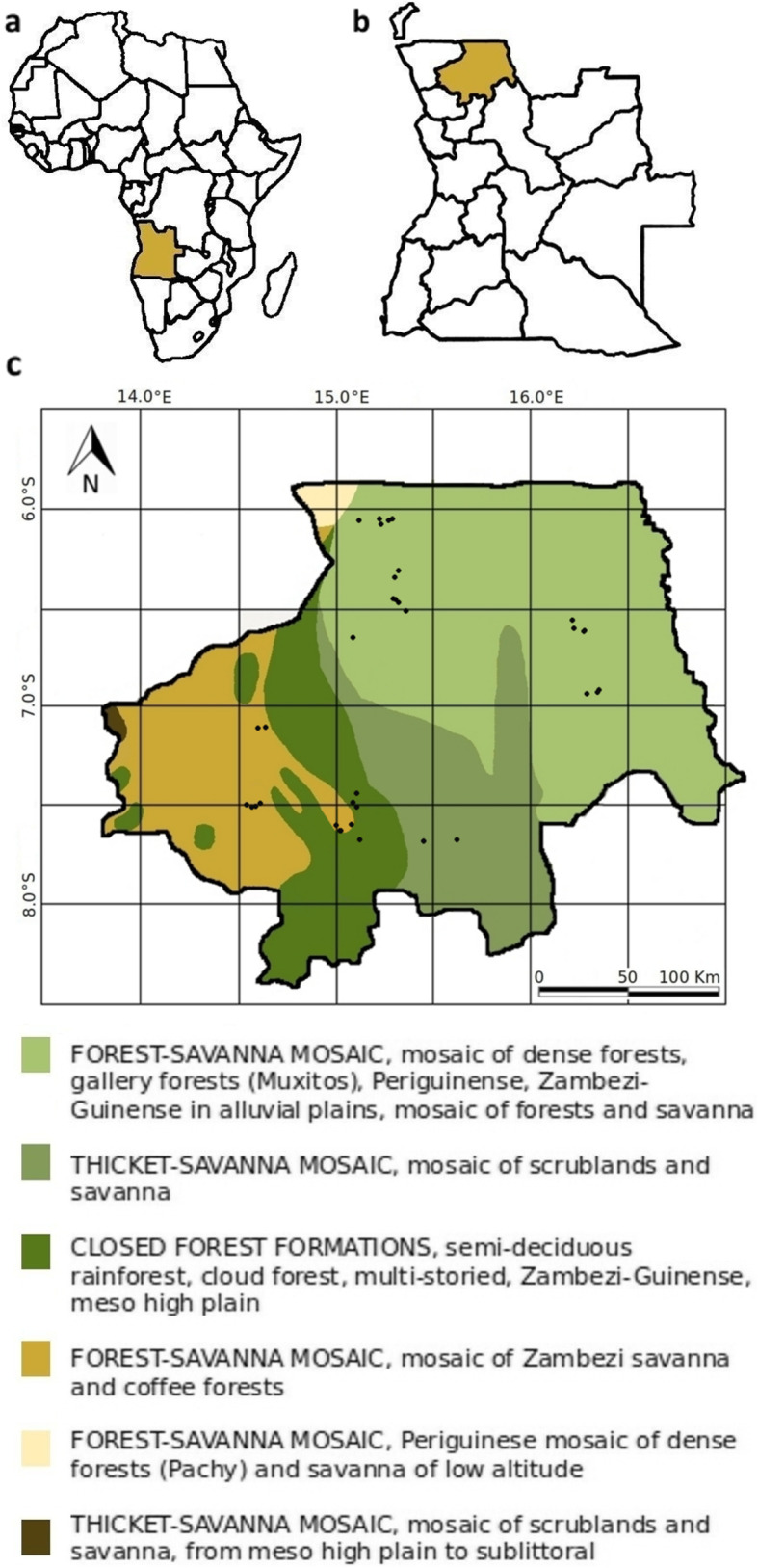
**a** Location of Angola in Africa **b** Province of Uíge in Angola **c** vegetation zones of the province Uíge and study locations marked with black dots; vegetation zones according to Barbosa [17]

The study was conducted in the north of Angola in the Province Uíge (Fig. 1). Uíge encompasses 16 municipalities. The study area covered the municipalities of Maquela do Zombo, Quimbele, Milunga, Damba, Bembe, Ambuila, Negage and Uíge. The coordinates of the study area are in the range of S 06°03.214′ to S 07°45.379′ and E 014°31.679′ to E 016°20.779′. The study area has a tropical wet and dry savannah climate (Köppen-Geiger classified as Aw) with temperatures above 18 °C and a dry season [18]. As shown in Fig. 1, most of the interview locations are in regions with a forest-savannah mosaic of dense or gallery forests and savannah. The province Uíge covers an area of 58,698 km^2^ and has a population of about 1,400,000 inhabitants, and population density of 24.3 inhabitants/km^2^ [19]. The majority of its population belongs to the BaKongo ethnic group.

### Data collection

The fieldwork took place in February and March 2018. Semi-structured interviews were conducted in 40 rural villages in eight municipalities of the Province Uíge. In total, 360 informants, aged between 16 and 81 years, representing 265 interviews were included, 96.1% of which were female, and 3.9% were male. Among the latter, 43% were interviewed together with women, while 29% were medical trained persons. Therefore, differences between the knowledge of men and women were not evaluated. Most of the female interview partners were mothers. Preferably, one-on-one interviews (216) were conducted, but group interviews (49) were also carried out. The group size varied from two to ten informants per group. Due to modifications of the interview structure during the study, not all questions were asked to each informant. Therefore, additional information on the extact number of informants are given, if necessary. The research included interviews with a doctor and a nurse from the “Hospital Provincial do Uíge” in the city Uíge, and three male nurses from the municipalities Ambuila, Quimbele and Bembe.

Sometimes the age of the informants had to be estimated. Most informants showed their identity card, because they did not know their exact age. If not available, the age was estimated. Some mothers did also have problems to make a statement about the age of their children. Therefore, all statements made about age and time spans should be treated carefully.

Prior to every interview, local authorities were informed about the project and asked for permission. On every field trip, at least one representative of the Universidade Kimpa Vita participated in the investigation. Interviews were carried out in Portuguese. If necessary, the co-workers or village residents translated from Portuguese into the traditional languages KiKongo or Lingala and vice-versa. The questions focussed on the knowledge on treatments. Women did not necessarily use these while breastfeeding.

When possible, plant samples were collected, or photos have been taken of every plant mentioned by the interviewees and identified at the Institute of Botany (Technische Universität Dresden). All specimens are stored in the Herbarium Dresdense (DR) and are available from Virtual Herbaria JACQ [20]. As soon as the required conditions are established, duplicates of the collection will be returned to Angola. The nomenclature refers to Tropicos, botanical information system at the Missouri Botanical Garden [21]. If a plant could not be identified, the local name is provided. The identified plants were categorised in endemic (E), naturalised (*), listed (+) and not listed (−) (Additional file 1). This categorisation is based on Figueiredo’s checklist “Plants of Angola” [22].

### Data analysis and ethnobotanical indices

To present the results of the women’s age, the number of births and the number of deceased children up to an age of five, we defined 11 categories, starting with 16 and ending with 81 because there were actually respondents at each end of that range. Six years are the optimal interval to categorize the numbers in each age group. Values are means, standard deviations (SD) are in brackets.

To determine the local importance of food and medicine mentioned by the informants, the Relative Frequency of Citations (RFC) was calculated (Formula 1). The RFC is the quotient of the division of the frequency of citations (FC) and the number of informants (*N*). As many interviews were conducted as group interviews, we used the total number of interviews per use category instead of the number of informants.

$$ {RFC}_s=\frac{FC_s}{N}=\frac{\sum \limits_{i={i}_1}^{i_N}{UR}_i}{N} $$

Formula 1: Calculation of the Relative Frequency of Citations (RFC): s = species, FC = Frequency of Citation by one informant; *N* = Total number of interviews per use category [23].

The data collection included four use categories. Plants, food, and treatments which (1) are used to increase lactation or promote breast milk production (Galactagogues); (2) are used to “clean” human breast milk; (3) should be avoided in the breastfeeding period; (4) are known to reduce human breast milk production. The term “cleaning” the breast milk is often used by women who have watery milk or assume that their breast milk is causing diarrhoea in their children.

The use of the plants recorded as galactagogues were compared with ethnobotanical literature including Neuwinger [10], Iwu [24], Kokwardo [25], Latham & Mbuta [26] and Mbuta [27].

## Results and discussion

### General aspects

#### Infant mortality rate

**Fig. 2:**
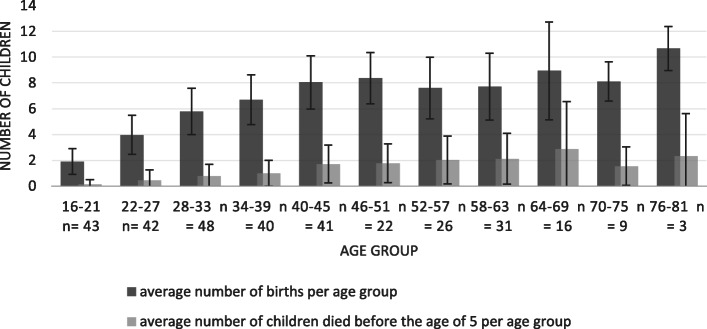
Average number of births and under-five mortality; *n* = number of informants. Error bars represent standard deviation (SD)

The interviewed mothers were between 16 and 81 years old. The number of births per mother varied from one to 21. The number of children rose from 1.9 births per mother in the age group of 16–21 years, to 8.4 births in women aging 46–51 years. Due to the onset of menopause around the age of 49 [28], the number of births did not increase beyond this age. The number of children that died before the age of five consistently increased from the first to the sixth age group. At the end of the reproductive phase, approximately 27% of children died before the age of five (Fig. 2). Not separately considered in this statistic are children who have not reached the age of five at the time of the interview. They are included in the average number of births. The average number of births over all age groups is 6.2 (± 3.0).

The data obtained hauntingly confirm the existing data on infant mortality in Angola. Albeit the official numbers show a declining trend, for the majority of rural people in the Province Uíge the health situation during the last decade did not change so that it remains open whether the decrease will also apply for northern Angola.

#### Duration of breastfeeding

The average duration of breastfeeding is 23 (± 5.2) months. Whenever the informants mentioned a time span for the termination of breastfeeding, the arithmetic average of this time span was used as the basis for the calculations. It was found that 52% of the interviewed women breastfed their children for 24 months, whilst 5.2% had a breastfeeding period of only 12 months, and 4.9% of 36 months. The reported lactation periods varied between 9 and 46 months.

Among those interviewed, 137 women provided information about the time they initiated normal food to the child’s diet in addition to breast milk. The average duration of exclusive breastfeeding was 6.1 (± 3.0) months.

Of the 304 women, 282 who made a statement about their breast milk production, did not have problems with their milk production (93%). Indeed, 126 women reported a high breast milk production whilst only 17 women mentioned the opposite. These results are supported by the statements given by the medically trained persons. In addition, with 23 months, the average duration of breastfeeding meets the recommendation of the WHO [7, 8] of 24 months or more. The children are exclusively breastfed for about 6 months again the time recommended by the WHO [7]. None of the women nursed their children less than 9 months. Breastfeeding is said to have many positive effects on the child’s health, especially in developing countries [9]. Breastfed children suffer considerably less from diseases like diarrhoea [29] or acute respiratory infections [30] and are less vulnerable to develop allergies [31] and chronical diseases like asthma [32] later in life, compared to children that are not breastfed in the first months of their lives.

For detected cases of child malnutrition, or when mothers have problems with their milk production, F75 therapeutic milk [33] is given in hospitals like the “Hospital Provincial do Uíge” in the city Uíge.

#### Transfer of traditional knowledge

The data highlight the important role the family plays in the transfer of the collected traditional knowledge. Virtually all women (97% of 99 asked women) received their information about the plants, food, and treatments affecting breast milk production by their family members. The majority of the interviewees (69%) acquired their knowledge from their mother, while 36% mentioned their grandmother as the person who provided the traditional knowledge. Only 4% of the women got knowledge from friends, acquaintances (2%), neighbours (1%), or elder people in the village (1%). A small percentage (2%) of women mentioned that they discovered the effect of specific treatments on their breast milk by themselves.

Not surprising, the family turned out to be of major importance for imparting traditional medical knowledge which is in accordance with other studies [34–36]. Especially the influence of mother and grandmother for knowledge transfer was previously recorded as well [34].

### Plants, foods and treatments with an impact on the human breast milk production

In total, 69 plant species (identified at least to genus level) and 21 other treatments were identified. Nineteen plants and remedies mentioned by interviewed people could not be identified, but are listed with their local KiKongo names in (Additional file 1: Tables S1-S4). The species belong to 36 plant families but predominantly from Fabaceae (14 species) and Euphorbiaceae (10 species). None of the identified plants is listed as endemic to the area, but 11 are naturalised [22]. Unless we focussed on plant species used to affect breast milk production, on occasion, the informants also recommended food and treatments, which were used. These are also listed and discussed in this study.

#### Increasing lactation

To increase or promote lactation, 39 plants (at least identified to genus level), 11 other edibles and medicines, and nine plants only known from their local name (Additional file 1: Table S1), were recorded in 61% of the 259 interviews. The 39 identified plants represent 25 different plant families.

In 57% of the interviews, *Manihot esculenta* was mentioned as a galactagogue followed by *Arachis hypogaea* L., (47%), and *Sesamum indicum* L. (12%). Fish (7%), *Elaeis guineensis* (7%), and *Saccharum officinarum* L. (5%) were as well mentioned more frequently compared to other plants, food, and medicine (Table 1).

**Table 1:** The most frequently named plants, foods, and treatments to increase lactation or promote breast milk production

Species	Common English name	Part	NC	RFC
*Manihot esculenta* Crantz	Cassava	L, R	148	0.568
*Arachis hypogaea* L.	Peanut	S	95	0.367
*Sesamum indicum* L.	Sesame	S	31	0.12
Fish	Fish		18	0.069
*Elaeis guineensis* Jacq.	Oil palm	S	17	0.066
*Saccharum officinarum* L.	Sugar cane	St	12	0.046

Listed in order of the number of citations (NC) and the Relative Frequency of Citation (RFC); Part: leaves (L), root (R), seeds (S), stem (St)

The treatments include a number of many different components, including the infusion of a bird’s nest, or the intake of minerals like salt. Furthermore, the decoction of a medical clay called Pemba, together with a bone, was recommended for women with poor breast milk production (Additional file 1: Table S1). In previous literature *Abrus precatorius* [26, 27], *Arachis hypogaea* [10], *Euphorbia hirta* [24–26], *Manihot esculenta* [10], *Milicia excelsa* [10, 24], and *Vitex doniana* [10, 24] have been reported as galactagogues.

The energy provided by most frequently cited plants and food can help the mother to meet the increased nutritional needs during lactation. The leaves and root tubers of *Manihot esculenta* are used as important food in sub-Saharan African communities [37]. The leaves are rich in protein, carotenoids, thiamine, riboflavin and minerals [38], whilst the root tuber is rich in starch [39]. The root is mostly consumed raw to promote or increase lactation. The plant produces linamarin, which can induce cyanide production. The leaves contain more linamarin than the root. Therefore, proper preparation is advised [40] and the long term consumption of a low level of linamarin can cause goitre or kinds of neuropathy [40, 41], but a good supply with S-containing amino acids is said to have a positive effect on the detoxification of cyanite in the body [41]. The occurrence of linamarin in cassava could be the reason why it was mentioned in a few interviews (3%) as food, which should be avoided during lactation.

The seeds of *Arachis hypogaea* are rich in fat (49 g) and protein (26 g). Of the total fat, 24 g in 100 g seeds are monounsaturated fatty acids, and around 16 g are polyunsaturated [42]. Among other minerals and vitamins, peanuts contain thiamine, riboflavin, folate and vitamin B6 [42] which are beneficial for the infant or the nursing mother [9]. Arya et al. (2016) stated that the fat of peanuts provides healthy calories to malnourished infants and children [43].

The seeds of *Sesamum indicum* also contain a high level of proteins and fatty acids most of which are polyunsaturated [44]. Sesame is the third most recommended galactagogue.

Contradictory statements exist about the effect of additional intake of proteins during lactation on the amount of protein in the breast milk [45]. However, the proteins present in the breast milk are produced in the mammary gland and in the blood of the mother [45]. The protein fraction of the mother’s milk additionally contains immunoglobulin, lactoferrin and lysozyme, which play an important role in the defence against pathogens [31]. The dominant carbohydrate in the breast milk is lactose, which is synthesised by the mother. Nevertheless, maternal intake of carbohydrates seems not to affect the concentration in the breast milk [45, 46].

The fat component is the most variable component of the breast milk and is related to the nutritional status of the mother. The milk of well-nourished mothers has a higher fat content than that of other mothers [45, 47]. It is widely proven that the maternal intake has an influence on the fatty acid composition of the breast milk [48]. To identify whether a woman’s breast milk has a good quality, four informants from different municipalities recommended to put a cockroach into a pot with breast milk to test the quality. If the cockroach is still alive after 5–30 min (the time varies depending on the informant) the milk has a good quality. There is no scientific explanation for it and we have not tested it either.

Only a small number of studies have been undertaken to examine the effect of herbal galactagogues on human breast milk production and the effect on mother and infant [15, 49]. Therefore, the effect for most galactagogues herbs on the breast milk production has not been proven, and reasons for the use of the identified plants remain speculative. Furthermore, herbals as well as chemical drugs should be used with caution during lactation to avoid overdosing and as a result to be protected against unwanted side-effects [50].

It is striking, that the galactagogues most frequently used by informants have a high nutritional value, but the effect of additional intake of carbohydrates and proteins even for malnourished women on the breast milk quantity is not adequately clarified.

#### Cleaning breast milk

As no equivalent scientific expression is available for the term “cleaning the breast milk” that is frequently used by women, we asked for a definition. According to the interviewees, the breast milk needs to be cleaned when it appears to be watery because this provokes diseases like diarrhoea.

In 220 interviews informants were asked about “milk-cleaning” plants, food, and treatments. We could identify 33 plant species that were thought to clean maternal milk. For three plants that could not be identified only the local name is available. *Spondias mombin* L. (36%), *Vernonia amygdalina* (11%), *Carica papaya* L. (7%), and *Elaeis guineensis* (7%) were the plants and medicines most frequently named in the interviews to “clean” aqueous or diarrhoea-causing breast milk (Table 2). *Carica papaya* and *Momordica charantia* L. are the only plants previously recorded as breast milk “cleaning” plants used by mothers in this area [51]. Additionally, Göhre et al. (2016) recorded *Euphorbia thymifolia* L. as a plant used by the local population to “clean” diarrhoea causing breast milk [52].

**Table 2:** The most frequently named plants, foods, and treatments to clean the breast milk production

Species	Common English name	Part	NC	RFC
*Spondias mombin* L.	Yellow mombin	L, B	80	0.364
*Vernonia amygdalina* Delile	Bitter leaf	L	24	0.109
*Carica papaya* L.	Papaya	L	15	0.068
*Elaeis guineensis* Jacq.	Oil palm	S	15	0.068
*Morinda morindoides* (Baker) Milne-Redh.	–	L	10	0.045
*Abrus precatorius* L.	Jequirity bean	L	9	0.046

Listed in order of the number of citations (NC) and the Relative Frequency of Citation (RFC); Part: leaves (L), bark (B), seeds (S)

The composition of breast milk varies over time and always fits the specific needs of the child. Especially the concentration of protein, fat, minerals and other components changes from the beginning to the end of the lactation period [31, 53]. The foremilk, which is secreted in the first minutes of every breast meal, has a high water content to quench the child’s thirst [31]. Therefore, this milk can be described as aqueous. Nevertheless, this milk should not cause diarrhoea. Several reasons may exist for diarrhoea in infants such as viruses or bacteria that can be easily transmitted by faecal residuals on surfaces, or by other people’s skin parts like the mother’s breast. In general, breast milk does not cause diarrhoea in infants. Other reasons for acute diarrhoea can be drugs, or poisons, or the immediate onset of hypersensitivity reactions. The reasons for chronic or persistent diarrhoea vary from parasite infections over food allergies, autoimmune disorders or other infections, to specific enzyme defects [5]. Malnutrition can contribute to the outbreak of diarrhoea [6].

Parts of *Spondias mombin* L., but especially the leaves, were mentioned in 36% of the interviews in relation to milk “cleaning” plants and in 3% of the interviews about galactagogues. In one interview, the participants did not recommend this plant for nursing mothers but in former studies, it had a positive effect on the initiation of breast milk secretion in West African dwarf (WAD) ewes [54]. The plant should not be used in high dosage over a long period, because it might cause hepatic and renal injury. This effect was reported in a study investigating the toxicological effects of *Spondias mombin* in adult male Wistar rats [55]. This finding can be related to the doctor’s statement that some women show signs of poisoning after they treated themselves with this plant. On the other hand, the leaf extract of *Spondias mombin* shows antimicrobial [56, 57] and antiviral activity [58]. In Nigeria, it is also used as a treatment for diarrhoea [59]. This might explain the high frequency of uses for this species to “clean” the breast milk.

The antimicrobial activity of some plants could explain the use of these plants for milk “cleaning” purposes. For instance, extracts of *Carica papaya* [60], *Spondias mombin* [56, 57], *Euphorbia hirta* [61] or *Azadirachta indica* A. Juss [62] show antimicrobial properties among others. Further studies are needed to explore whether some of their antimicrobial substances are transferred in the breast milk and have an antimicrobial effect against several pathogens inside the infant.

#### To avoid

Complementary to the former aspects of treatments in 236 interviews the informants were asked about plants, food, and treatments, which should be avoided during breastfeeding periods. In 42% of the interviews, in total 32 plants (29 identified, three unidentified) and 11 other food and habits were mentioned that should be avoided during lactation period (Additional file 1: Table S3).

Dried fish was named in 16.1% of the interviews, followed by *Mangifera indica* L. (7.2%), and *Salacia erecta* (G.Don) Walp. (5.1%, Table 3). Dried fish was often described to cause watery milk and diarrhoea in children. In fact, many of the plants, foods, and treatments described were assumed to cause watery milk and diarrhoea in the child. According to the information provided, breast milk will dry up when the mother consumes *Monodora myristica* (Gaertn.) Dunal or *Salacia erecta*.

**Table 3:** The most frequently named plants, foods, and treatments which should not be eaten or applied during lactation period

Species	Common English name	Part	NC	RFC
Fish	Fish		38	0.161
*Mangifera indica* L.	Mango	F	17	0.072
*Salacia erecta* (G.Don) Walp.	–	L	12	0.051
*Elaeis guineensis* Jacq.	Oil palm	F, O	10	0.042
Goat	Goat		10	0.042
*Cucurbita* spec.	Pumpkin	L, F	9	0.038

Listed in order of the number of citations (NC) and the Relative Frequency of Citation (RFC); Part: fruit (F), leaves (L), oil (O)

Similarly rated was the consumption of goat meat supposedly having a negative effect on the child, eventually causing its death. In addition, the consumption of many animal products like eggs, pork or the meat of antelope, birds, chicken, goat, or fish was not recommended for lactating women as well as removing birds’ nests and the consumption of bitter plants, salt, or meat with vegetables.

Several studies showed, that phytochemicals can be transmitted from the mother to the child by breast milk and have an effect on the infants’ health [63, 64]. However, phytochemicals can also be toxic for other organisms than for the producing one – and thus also for humans [65]. Nevertheless, only few adequate studies are available, which observe the risk of herbal treatments during lactation for the infants health [15, 49, 66]. The plants mentioned in this study are probably avoided because they also reduce milk production (e.g. *Monodora myristica*, *Salacia erecta*). The prohibition of the consumption of several foods and plants can also have traditional reasons explained later.

#### Reduce lactation

In 124 interviews, the informants were asked about information on plants, foods, or treatments, which are used to reduce the breast milk production. The informants recommended 17 identified plant species, 3 unidentified plants, 4 habits and the consumption of fish (Additional file 1: Table S4).

*Monodora myristica* was named in three interviews (2.4%) and is therefore the most commonly used remedy for breast milk reduction. Other common remedies are *Abrus precatorius* L., *Saccharum officinarum*, and *Salacia erecta* (Table 4).

**Table 4:** The most frequently named plants, foods and treatments to reduce the breast milk production

Species	Common English name	Part	NC	RFC
*Monodora myristica* (Gaertn.) Dunal	Calabash nutmeg	S	3	0.024
*Abrus precatorius* L.	Jequirity bean	L	2	0.016
*Saccharum officinarum* L.	Sugar cane	St	2	0.016
*Salacia erecta* (G.Don) Walp.	–	L	2	0.016
Clean the body	–		2	0.016
Reduced food intake	–		2	0.016

Listed in order of the number of citations (NC) and the Relative Frequency of Citation (RFC); Part: seeds (S), leaves (L), stem (St)

Common herbs, which are used to decrease lactation in other countries, are peppermint, sage and parsly [50]. In only 19% of the interviews, respondents recommended plants, food, or treatments at all. *Monodora myristica*, the plant that mentioned most frequently, is only recommended in 2.4% of the interviews. This indicates the minor importance of plants, foods, and treatments to reduce breast milk production. Additionally, the four most recommended plants are also mentioned in other use categories but with higher frequency. In 1.6% of the interviews, a reduced food intake was recommended to decrease lactation. A decrease of milk in animals by reducing the energy supply could be documented in some studies, but the reduction of energy intake in lactating mothers is not adequately investigated yet [53].

#### Ethnological perspective

Little research has been conducted with respect to the cultural habits of the local population in the study area. An important exception is Laman’s study of the Bakongo people in the Lower Congo region. In his research, although concentrating on an area outside of our study region, Laman reports many cultural habits of the BaKongo people in the Lower Congo region [67–69]. As an example, women lacking milk production after birth drink a mixture that includes nzeke-nzeke [68]. This plant most probably is similar to *Craterispermum* spec., in our study called nseka-seka, and identified inter alia for increasing or promoting lactation. The consumption of salt, pepper and other tasty things is also recommended for women with lactation problems [68]. In two interviews, salt was mentioned to promote or increase lactation. Additionally, five galactagogue plants were suggested to be taken or prepared together with salt. In contrast, Laman also notes that coarse salt should not be eaten by nursing mothers because the milk will be curdled and the child will fall ill [69]. Compared with our findings, salt is more often recommended to increase or promote lactation, instead of the opposite.

Due to prohibitions which are part of the BaKongo culture, several plants, foods, and treatments are forbidden for a certain person or a group of people [67–69]. However, in other cultures, restrictions especially for nursing mothers exist as well [70, 71].

Pork, for instance, plays a special role in the local culture. The meat is prohibited for many people because it is assumed to have negative effects on the body. For example, it is said that if the mother eats pork, the child will fall ill [69]. While in one interview, pork was reported as a food that should be avoided during the breastfeeding period, three other persons, recommended pork as a galactagogue. In fact it does have nutritional benefits for the nursing mother because of its high content in protein, fat, iron, zinc and B vitamins like Thiamin [72], but can also be contaminated by parasites, which has a negative effect on the mothers’ well-being [73].

Laman documents, that the Nkabi-antelope or other striped or spotted animals are often banned for women or certain families as they are said to cause ringworm rash or similar eruptions [67]. He also notes that the consumption of eggs during pregnancy is forbidden for both the wife and her husband because it will affect the appearance of the child. The consumption of goat and chicken is also not recommended for pregnant women. Generally, for women it is often forbidden to eat the best parts of mammals and fish [67].

Some of these traditional nutritional taboos may have been preserved and influence the eating habits of the Bakongo people today. This could explain why the consumption of pork, salt, patterned antelope, eggs, and feathered animals like chicken are not recommended for consumption during breastfeeding, which was noted in some interviews. The most common food restriction was the abdication of eating fish noted in 16% of the interviews. In a survey conducted by Kouser Banu et al. (2016) in Tamilnadu (India), fish is also reported as a prohibited food during pregnancy and lactation [74]. The consumption of fish does have some risk, especially if the fish is not fresh or prepared properly, or is contaminated with bacteria during processing. On the other hand, fish is rich in Group 1 micronutrients like vitamin D, iodine and riboflavin and several Group 2 micronutrients like iron, calcium and zinc [75]. The amounts of Group 1 micronutrients in the breast milk depend on the intake of these nutrients by the mother. The secretion of Group 2 nutrients into the mother’s milk does not depend on the dietary intake of the mother, but a maternal supplementation with these nutrients is beneficial for her [75]. Therefore, it is not surprising that fish is also the fourth most common galactagogue recommended in this study (Table 2).

Other documented traditions, like the ban on listening to the wind soughing in bunches of bananas and melon-tree [69] to prevent the milk from coagulation or child illness, or the consumption of tiba-bananas and binsakulu-tomatoes by the mother along with others, were not mentioned by our informants.

Laman’s research provides an insight into traditional uses of plants, foods, and treatments. However, the recorded prohibitions and food regulations in this study could also have other reasons. Some plants have names, which are related to their usage or their characteristics. For example, “Kimvumina” is the KiKongo word for mother’s milk but also the name of several plants such as *Gongronema latifolium* Benth. and *Euphorbia hirta* L.. The naming may have its origin in the production and secretion of a white milk sap that is strikingly similar to regular milk, or due to its effect on breast milk production. The use of *Euphorbia hirta* as a galactagogue was recorded once in our study but also known from previous studies [11, 24, 26] and mentioned as a milk “cleaning” plant as well. The latter application could be related to its antidiarrhoeic activity [76] and antibacterial effects against some gram-positive bacteria, causing enteric infections [61]. *G. latifolium* is used in the same way for both as a galactagogue and to “clean” the breast milk. The leaves can be used as a source for important nutrients like proteins [77] and especially the ethanolic extract of the leaves again has shown antimicrobial activity against some bacteria [77, 78].

The use of *Azadirachta indica* as a milk “cleaning” plant may also be related to its name. Its Portuguese name is “Curatudo”, which can be translated as a cure-all. Therefore, it might be used by local people for many health-related problems. Indeed, *Azadirachta indica* is used by many people historically and today for many different applications [79]. This includes the treatment of diarrhoea and cholera by Indian people [80], diabetes in Bali (Indonesia) [81], or stomach pains by locals in the province Uíge in Angola [51]. The leaf extract has shown antimicrobial activity [62] even against some multi-resistant microorganisms [82].

#### Qualitative analysis of statements made by medical staff

The interviews included four with nurses, and one with a doctor. All medical persons had completed a professional medical training. The nurses stated that the medical resources at their disposal were not sufficient. For several diseases like malaria, they have neither medical tests, nor a microscope for simple testing. They do have the authorisation to prescribe medication and tests. The nurses did not see a problem in the use of traditional medicine, and even knew several of them. The interviewed doctor in the hospital in Uìge, however, saw problems with the traditional treatments applied by women. He experienced cases of slightly poisoned children after the consumption of *Spondias mombin* leaves. He noted that the wrong dosage of medicinal plants can cause many negative effects on the mother and the child.

The nurses stated that women do not have many problems with lactation. The doctor supported this assessment. He said that most women breastfeed their children very often and over a long period. Furthermore, in cases when the mother does not have enough breast milk, this is often caused by a chronic disease such as hepatitis, or other diseases like breast cancer or mastitis. In these cases, the infant receives a therapeutic breast milk substitute, such as F75 [33], which was mentioned by the nurse in the hospital. One male nurse recommended Amodiaquin to clean the breast milk. Amodiaquin is a remedy used as a treatment against malaria. The informant testified that malaria is the most common cause of child death in this region.

There is a clear difference between the interviews conducted with the hospital staff members and the local nurses in the villages. The doctor pointed out that the nutrient supply of children is deficient, while for the nurses in the villages malnutrition is not a major problem in this area of the country.

The doctor stated that an unbalanced diet of the mother has an impact on breast milk quality. This can lead to the malnutrition of children who consequently suffer from many diseases that eventually are lethal. Additionally, many childbirths take place in unsanitary conditions, which also has an impact on the high child mortality rate. According to the doctor, a lack of prenatal examinations leads to many children with disabilities.

## Conclusions

Breastfeeding is an indispensable for the life of children in the province Uíge and traditional knowledge about plants, foods, and treatments, which affect the breast milk production, is widespread. This study shows that the majority of the women in the province Uíge do not have any problems with their breast milk production and nurse their children for a long period. However, malnutrition seems to be a problem in this area and can be a major factor for the high under-five mortality rate. The informants knew the most about plants, foods, and treatments, which clean the breast milk and only a little less about natural galactagogue whilst many plants are named for both uses. The cultural background of the informants seems to have an influence on the knowledge and habits recorded. This study gives an overview over the plants, foods, and treatments used to affect the quality and quantity of the human breastmilk by the local BaKongo population. Further studies about the safety and efficiency of the recommended plants, foods and treatments could be conducted to support their use by lactating mothers.

## Supplementary information

**Additional file 1: Table S1.** Overview of plants, foods and treatments which increase or promote lactation. **Table S2.** Overview of plants, foods and treatments which are mentioned for “cleaning” the breast milk. **Table S3.** Overview of plants, foods and treatments a lactating mother should not use. **Table S4.** Overview of plants, foods and treatments which are used to decrease the breast milk production.

**Additional file 2:.** Locations of the villages where the interviews were conducted.

## Data Availability

All data are available from the corresponding author. All voucher specimens are deposited in the Herbarium Dresdense (DR) of the Institute of Botany, Technische Universität Dresden, Germany. As soon as suitable conditions are established, parts of the collection will be deposited at University Kimpa Vita, Uíge, Angola.

## Abbreviations

RFC: Relative Frequency of Citations
UNICEF: United Nations Children’s Fund
WHO: World Health Organisation
