# Supplementary material for: Plants, food and treatments used by BaKongo tribes in Uíge (northern Angola) to affect the quality and quantity of human breast milk
Source: Int Breastfeed J. 2020 Oct 23;15:88. doi: 10.1186/s13006-020-00329-1 (PMC7583195; doi:10.1186/s13006-020-00329-1)
Supplement: Supplementary file 1 — Additional file 1: Table S1. Overview of plants, foods and treatments which increase or promote lactation. Table S2. Overview of plants, foods and treatments which are mentioned for “cleaning” the breast milk. Table S3. Overview of plants, foods and treatments a lactating mother should not use. Table S4. Overview of plants, foods and treatments which are used to decrease the breast milk production. [file 13006_2020_329_MOESM1_ESM.docx]

Table 5: Overview of plants, foods and treatments which increase or promote lactation. All identified plant species were categorised in endemic (E), naturalised (*), listed (+) and not listed (-) (Neuwinger 2000). For most plants the herbarium number according to Herbarium Dresdense (HD) is listed. In cases where no herbarium specimen was collected the number of a photo proof (F or G-F) is given. Plants which were identified with the comparison of the Portuguese or Kikongo names with the data collection of Lautenschläger (2018)^19^ were marked with an •. To verify the use as a galactagogue the following references were checked: 1 = Neuwinger (2000); 2 = Iwu (2014); 3 = Latham (2014); 4 = Konda ku Mbuta (2012); 5 = Kokwardo (2009). If the literature states another use than as a galactagogue it is shown by a “- “. A ”/” points out that no information about this plant is given. Only plants used as a galactagogue as recorded are marked with a “+”. If the plant is used as a galactagogue but there is another part of the plant or another preparation cart in the literature this is shown by an “o”. part: b = bark, ba = bacon, bo = bone, c = coconut milk, cl = clay, f = fruit, l = leaves, r = root, s= seed, ss = stem sap, st = stem; n = no further information; Total number of citations in the interviews (CI), number of interviews (N), KiKongo name (Kik), Portuguese name (Port.)

| **Scientific name** | **Herbarium number HD** | **Family** | **Local name** | **Part** | **Preparation** | **Citations** | **CI** | **RFC** (N = 259) | **References** |
| --- | --- | --- | --- | --- | --- | --- | --- | --- | --- |
| *^+^Abrus precatorius* L. | 051649 | Fabaceae | Maique, Kiambiembie (Kik.) | r |  | 1 | 3 | 0,012 | -^1^; -^2^; +^3^; o^4^; -^5^; |
|  |  |  |  | l |  | 2 |  |  |  |
| *^+^Aframomum alboviolaceum* (Ridl.) K. Schum. | 051614 | Zingiberaceae | Gingenga da queimada (Port.) | f |  | 1 | 1 | 0,004 | -^1^; /^2^; -^3^; /^4^; /^5^; |
| *^*^Amaranthus caudatus* L. | 051616 | Amaranthaceae | Gimboa, Bowa (Kik.) | l |  | 2 | 4 | 0,012 | -^1^; -^2^; /^3^; -^4^; -^5^; |
|  |  |  |  | st |  | 1 |  |  |  |
|  |  |  |  | n |  | 1 |  |  |  |
| *^*^Arachis hypogaea* L. | G-F10 | Fabaceae | Ginguba (Kik.) | s | fresh | 54 | 95 | 0,367 | +^1^; -^2^; -^3^; -^4^; /^5^; |
|  |  |  |  |  | any form | 3 |  |  |  |
|  |  |  |  |  | With salt (dermal) | 1 |  |  |  |
|  |  |  |  |  | n | 37 |  |  |  |
| *Brassica* spec.^•^ | 042794 | Brassicaceae | Couve (Port.), Nkove (Kik.) | l | decoction | 2 | 10 | 0,039 | *Brassica integrifolia* (root) +^1^; *Brassica juncea* (leaves, oil) +^2^; -^3^; /^4^; *Brassica carinata* +^5^; |
|  |  |  |  |  | raw | 1 |  |  |  |
|  |  |  |  |  | n | 7 |  |  |  |
| *^+^Brillantaisia owariensis* P.Beauv. | 051631 | Acanthaceae | Lemba Lemba (Kik.) | l | infusion | 1 | 1 | 0,004 | -^1^; -^2^; -^3^; -^4^; /^5^; |
| *^*^Carica papaya* L. | F_22 | Caricaceae | Mamão (Port.), Papayi (Kik.) | f |  |  | 1 | 0,004 | -^1^; -^2^; -^3^; -^4^; /^5^; |
| *^*^Cocos nucifera* L. | G-F1  G-F2 | Arecaceae | Coco (Port.) | f | With Kokonote (*Elaeis guineensis*) | 1 | 3 | 0,012 | -^1^; -^2^; -^3^; /^4^; /^5^; |
|  |  |  |  | c |  | 2 |  |  |  |
| *^+^Combretum* *racemosum* P.Beauv. | 053487 | Combretaceae | Nsumbisila, Nsumbila (Kik.) | n |  |  | 1 | 0,004 | -^1^; -^2^; /^3^; -^4^; /^5^; |
| *^+^Costus afer* Ker Gawl. | 051612 | Costaceae | Mikeni (Kik.) | n |  |  | 1 | 0,004 | -^1^; -^2^; -^3^; -^4^; /^5^; |
| *Costus* spec. | 051608  053482 | Costaceae | Nsangalavua (Kik.) | st | With salt | 2 | 4 | 0,015 | *Costus lucanisianus* (twig sap) +^1^; *Costus lucanisianus* (leaves sap, aerial parts) +^2^; -^3^; -^4^; -^5^; |
|  |  |  |  |  | With Mandioca (*Manihot esculenta*) and Kokonote (*Elaeis guineensis*) |  |  |  |  |
|  |  |  |  | n |  | 2 |  |  |  |
| *Craterispermum* spec. | 053476 | Rubiaceae | Kiseka seka, Nseka-seka (Kik.) | r | Eat with raw Mandioca (*Manihot esculenta*) and Ginguba (*Arachis hypogaea*) | 1 | 3 | 0,012 | -^1^; -^2^; -^3^; -^4^; -^5^; |
|  |  |  |  | b | eat, chew | 2 |  |  |  |
| *Cucurbita* spec. |  | Cucurbitaceae | Muteta, Abóbora (Port.), Muengeleka (Kik.) | s | decoction | 1 | 6 | 0,023 | -^1^; -^2^; -^3^; /^4^; /^5^; |
|  |  |  |  |  | Grind and prepare like a small cake | 3 |  |  |  |
|  |  |  |  | l |  | 1 |  |  |  |
|  |  |  |  | n |  | 2 |  |  |  |
| *^+^Elaeis guineensis* Jacq. | G-F3  G-F4  G-F5 | Arecaceae | Kokonote (Kik.) | s | Raw | 1 | 17 | 0,066 | -^1^; o^2^; -^3^; -^4^; -^5^; |
|  |  |  |  |  | With Mandioca (*Manihot esculenta*) | 1 |  |  |  |
|  |  |  |  |  | With coconut | 1 |  |  |  |
|  |  |  |  |  | With Mandioca (*Manihot esculenta*) and Sangalavua (*Costus* spec.) | 1 |  |  |  |
|  |  |  |  |  | n | 13 |  |  |  |
| *^*^Euphorbia hirta* L. | 051624  051625 | Euphorbiaceae | Kimvumina, Kimvumina kia nkombo (Kik.) | l | maceration | 1 | 1 | 0,004 | o^1^; +^2^; +^3^; -^4^; o^5^; |
| *^+^Gongronema latifolium* Benth. | 051613 | Apocynaceae | Kimvumina (Kik.) | n |  |  | 4 | 0,015 | -^1^; -^2^; /^3^; /^4^; /^5^; |
| *^+^Heinsia crinita* (Afzel.) G.Taylor | 053486  051950 | Rubiaceae | Nsangumuni (Kik.) | n |  |  | 1 | 0,004 | -^1^; -^2^; -^3^; -^4^; -^5^; |
| *^*^Jatropha curcas* L. | 051620 | Euphorbiaceae | Mpuluka (Kik.) | l | Infusion | 1 | 2 | 0,008 | -^1^; o^2^; -^3^; -^4^; -^5^; |
|  |  |  |  |  | maceration | 1 |  |  |  |
| *^*^Mangifera indica* L. | 42871 | Anacardiaceae | Manga (Port.) | n |  |  | 1 | 0,004 | -^1^; -^2^; -^3^; -^4^; /^5^; |
| *^-^Manihot esculenta* Crantz | 42760 | Euphorbiaceae | Mandioca (Port.), Funge, Kisaka, N´saki (Kik.) | l | Raw | 1 | 147 | 0,568 | +^1^; /^2^; -^3^; -^4^; /^5^; |
|  |  |  |  |  | n | 45 |  |  |  |
|  |  |  |  | r | Raw | 56 |  |  |  |
|  |  |  |  |  | cooked | 1 |  |  |  |
|  |  |  |  |  | Row with Coconote (*Elaeis guineensis*) | 1 |  |  |  |
|  |  |  |  |  | n | 41 |  |  |  |
|  |  |  |  |  | Mash (Funge) | 2 |  |  |  |
| *^+^Maprounea africana* Müll.Arg. | 051640 | Euphorbiaceae | Nsiele nsiele (Kik.) | l | raw | 1 | 2 | 0,008 | -^1^; -^2^; /^3^; /^4^; -^5^; |
|  |  |  |  |  | n | 1 |  |  |  |
| *^+^Milicia excelsa* (Welw.) C.C.Berg | 051637 | Moraceae | Nkamba (Kik.), Moreira (Port.) | st | maceration | 1 | 3 | 0,012 | +^1^; +^2^; -^3^; -^4^; -^5^; |
|  |  |  |  | b | maceration | 1 |  |  |  |
|  |  |  |  | ss |  | 1 |  |  |  |
| *^+^Momordica charantia* L. | 051646 | Cucurbitaceae | Lumbuzua, Mambuzu (Kik.) | l | maceration | 1 | 2 | 0,008 | -^1^; -^2^; -^3^; /^4^; /^5^; |
|  |  |  |  | n |  | 1 |  |  |  |
| *Musa* spec. |  | Musaceae | Banana (Port.) | n |  |  | 1 | 0,004 | -^1^; -^2^; -^3^; -^4^; -^5^; |
| *^+^Periploca nigrescens* Afzel. | 053481 | Apocynaceae | Lombua, Malombua (Kik.) | b | Raw | 1 | 1 | 0,004 | -^1^; -^2^; /^3^; -^4^; /^5^; |
|  |  |  |  |  | maceration |  |  |  |  |
| *^-^Persea americana* Mill. |  | Lauraceae | Abacate (Port.) | l |  | 1 | 3 | 0,012 | -^1^; /^2^; -^3^; -^4^; /^5^; |
|  |  |  |  | n |  | 2 |  |  |  |
| *^*^Phaseolus vulgaris* L.^•^ | 42758 | Fabaceae | Feijões, (Port.)  Makasikila (Kik.) | s |  | 2 | 4 | 0,015 | /^1^; /^2^; -^3^; /^4^; /^5^; |
|  |  |  |  | l |  | 2 |  |  |  |
| *^+^Psophocarpus palustris* Desv. | 051638 | Fabaceae | Kikalakassa, Mpavu (Kik.) | l | Cooked and eaten with salt | 1 | 1 | 0,004 | -^1^; /^2^; / ^3^; /^4^; /^5^; |
| *^+^Psorospermum febrifugum* Spach | 051609 | Hypericaceae | Fiofio (Kik.) | l |  |  | 1 | 0,004 | -^1^; -^2^; -^3^; /^4^; -^5^; |
| *^+^Raphia matombe* De Wild. | 050857 | Arecaceae | Maruvo (Kik.) |  |  |  | 2 | 0,008 | /^1^; /^2^; -^3^; /^4^; /^5^; |
| *^*^Saccharum officinarum* L*.* |  | Poaceae | Cana de açúcar (Port.) | st |  |  | 12 | 0,046 | -^1^; -^2^; -^3^; /^4^; /^5^; |
| *^+^Sesamum indicum L.* | 043895 | Pedaliaceae | Gergelim (Port.), Wanguila (Kik.) | s | With Fungi | 1 | 31 | 0,120 | -^1^; o^2^; -^3^; /^4^; /^5^; |
|  |  |  |  |  | Raw and cooked | 1 |  |  |  |
|  |  |  |  |  | Cooked and eaten in food | 2 |  |  |  |
|  |  |  |  |  | n | 27 |  |  |  |
| *^+^Solanum macrocarpon* L.^•^ | 044099  G-F6 | Solanaceae | Couve preta (Port.) | l | decoction | 1 | 2 | 0,008 | -^1^; -^2^; -^3^; /^4^; /^5^; |
|  |  |  |  |  | n | 1 |  |  |  |
| *^*^Spondias mombin* L. | 051647 | Anacardiaceae | Mungiengie (Kik.), Mingiengie (Kik.),  Gajajeira (Port.) | l | masticate raw | 1 | 7 | 0,027 | -^1^; -^2^; -^3^; /^4^; /^5^; |
|  |  |  |  |  | infusion | 1 |  |  |  |
|  |  |  |  |  | n | 3 |  |  |  |
|  |  |  |  | r | n | 1 |  |  |  |
|  |  |  |  | n |  | 2 |  |  |  |
| *^+^Terminalia brachystemma* Welw. ex Hiern | 051607 | Combretaceae | Mungolo (Kik.) | r |  |  | 1 | 0,004 | -^1^; /^2^; /^3^; /^4^; /^5^; |
| *^+^Uvaria poggei* Engl. & Diels^•^ | 050910 | Annonaceae | Nkombo (Kik.) | n |  |  | 1 | 0,004 | -^1^; /^2^; /^3^; /^4^; /^5^; |
| *^+^Vernonia amygdalina* Delile | 051610 | Asteraceae | Malulu, Malulua (Kik.) | l | Maceration | 2 | 3 | 0,012 | -^1^; -^2^; -^3^; -^4^; -^5^; |
|  |  |  |  |  | masticate raw | 1 |  |  |  |
| *^+^Vitex doniana* Sweet | 053483 | Lamiaceae | Balafilo (Kik.) | n |  |  | 1 | 0,004 | +^1^; +^2^; -^3^; /^4^; -^5^; |
| *^*^Zea mays* L. |  | Poaceae | Milho (Port.) | s | Decoction with salt | 1 | 3 | 0,012 | -^1^; -^2^; -^3^; -^4^; /^5^; |
|  |  |  |  |  | Raw | 1 |  |  |  |
|  |  |  |  |  | n | 2 |  |  |  |
| Bird´s nest |  |  | Ninho de pássaros (Port.) |  | infusion |  | 1 | 0,004 |  |
| Eggs |  |  | Ovos (Port.) |  |  |  | 2 | 0,008 |  |
| Evaporated milk |  |  |  |  |  |  | 1 | 0,004 |  |
| Fish |  |  | Peixe, peixe seco (Port.) |  | Fresh | 1 | 18 | 0,069 |  |
|  |  |  |  |  | Dried | 17 |  |  |  |
| Meat |  |  |  |  |  |  | 1 | 0,004 |  |
| Milk |  |  | Vaca (Port.) |  |  |  | 2 | 0,008 |  |
| Minerals, Salt |  |  | Minérios, Sal (Port.) |  |  |  | 2 | 0,008 |  |
| Pork |  |  | Porco (Port.) | ba | n | 1 | 3 | 0,012 |  |
|  |  |  |  | bo | Decoction | 2 |  |  |  |
| Vegetables |  |  | Verduras (Port.) |  | Without meat | 1 | 2 | 0,008 |  |
|  |  |  |  |  | n | 1 |  |  |  |
| Mixture |  |  | milk, mgomagome (bark), Minkombo (root), Kapidi (*Piper guineense*), Mpeve (*Monodora myristica*), Nsaku (root), Manga (*Mangifera indica*) (bark) |  |  |  | 1 | 0,004 |  |
| mushroom |  |  | Utunturuo (Kik.), Kukumelo (Port.) |  |  |  | 1 | 0,004 | mushroom |
|  |  |  | Kimvumina, Kimvumina kia nkombo (Kik.) |  |  |  | 1 | 0,004 |  |
|  |  |  | Manquila, Maquila (Kik.) |  |  |  | 2 | 0,008 |  |
|  |  |  | Mungimba (Kik.) | r |  |  | 1 | 0,004 |  |
|  |  |  | Munzenzenzenze (Kik.) | l | Raw |  | 1 | 0,004 |  |
|  |  |  | Ngubanguba (Kik.) | l | infusion |  | 1 | 0,004 |  |
|  |  |  | Nkambiebie (Kik.) | l | maceration |  | 1 | 0,004 |  |
|  |  |  | Ntenda (Kik.) | r | With Mandioca (*Manihot esculenta*) | 1 | 2 | 0,008 |  |
|  |  |  |  | n |  | 1 |  |  |  |
|  | F8, F9, F10 |  | Mpemba, Luvemba, Mabele (Kik.) | cl | Decoction with a bone |  | 1 | 0,004 |  |
|  |  |  | Usa | l | Decoction and eaten with Ginguba (*Arachis hypogaea*) and salt |  | 1 | 0,004 |  |

Table 6: Overview of plants, foods and treatments, which are mentioned for “cleaning” the breast milk. All identified plant species were categorised in endemic (E), naturalised (*), listed (+) and not listed (-) (Neuwinger 2000). For most plants, the herbarium number according to Herbarium Dresdense (HD) is listed. In cases where no herbarium specimen was collected the number of a photo proof (F or G-F) is given. Plants, which were identified with the comparison of the Portuguese or Kikongo names with the data collection of Lautenschläger (2018) were marked with an •; part: b = bark, ba = bacon, bo = bone, cl = clay, f = fruit, l = leaves, o = oil, p = whole plant, r = root, s= seed, ss = stem sap, st = stem; n = no further information; Total number of citations in the interviews (CI), number of interviews (N), KiKongo name (Kik), Portuguese name (Port.)

| **Scientific name** | **Herbarium number** | **Family** | **Local name** | **Part** | **preparation** | **Citations** | **CI** | **RFC** (N = 220) |
| --- | --- | --- | --- | --- | --- | --- | --- | --- |
| *^+^Abrus precatorius* L. | 051649 | Fabaceae | Kiambiembie (Kik.) | l | Chewed | 6 | 9 | 0,041 |
|  |  |  |  |  | Maceration | 2 |  |  |
|  |  |  |  |  | n | 1 |  |  |
| *^+^Albizia adianthifolia* (Schum.) W.Wight | 051615 | Fabaceae | Mulu (Kik.) | n |  |  | 1 | 0,005 |
| *^*^Arachis hypogaea* L. |  | Fabaceae | Ginguba (Kik.) | s | Row | 1 | 2 | 0,009 |
|  |  |  |  |  | n | 1 |  |  |
| ^-^*Azadirachta indica* A.Juss. | 051636 | Meliaceae | Curatudo (Port.) | l | Maceration | 1 | 2 | 0,009 |
|  |  |  |  | n |  | 1 |  |  |
| *^+^Bauhinia thonningii* Schum.^•^ | 043847 | Fabaceae | Loloa (Kik.) | r | Maceration | 1 | 2 | 0,009 |
|  |  |  |  | l | Maceration | 1 |  |  |
| *^*^Carica papaya* L. | F_22 | Caricaceae | Mamão (Port.), Papayi (Kik.) | l | Maceration | 11 | 15 | 0,068 |
|  |  |  |  |  | n | 4 |  |  |
| *^+^Cayratia gracilis* (Guill. & Perr.) Suess. | 053475 | Vitaceae | Lembozi (Kik.) | l | Maceration | 1 | 2 | 0,009 |
|  |  |  |  | n |  | 1 |  |  |
| *^*^Cocos nucifera* L. | G-F1  G-F2 | Arecaceae | Coco (Port.) | f |  |  | 4 | 0,018 |
| *Craterispermum* spec. | 053485  053484 |  | Nseka seka (Kik.) | n |  |  | 1 | 0,005 |
| *^+^Dacryodes edulis* (G.Don) H.J.Lam | F_37  G-F7 | Burseaceae | N´safu (Kik.) | b | Maceration |  | 1 | 0,005 |
| *^-^Dysphania ambrosioides* (L.) Mosyakin & Clemants | 051634 | Amaranthaceae | Santa Maria (Port.) | n | With raw Mandioca (*Manihot esculenta*) |  | 1 | 0,005 |
| *^+^Elaeis guineensis* Jacq. | G-F3  G-F4  G-F5 | Arecaceae | Kokonote (Kik.) | s |  |  | 15 | 0,068 |
| *^+^Entada abyssinica* A.Rich. | 051627 | Fabaceae | Nsofi (Kik.) | l | Raw |  | 1 | 0,005 |
| *^*^Euphorbia hirta* L. | 051624  051625 | Euphorbiaceae | Kimvumina, Kimvumina kia nkombo (Kik.) | l | With Kokonote (*Elaeis guineensis*) | 1 | 1 | 0,005 |
| *^+^Gongronema latifolium* Benth. | 051613 | Apocynaceae | Kimvumina (Kik.) | st | Maceration | 2 | 5 | 0,023 |
|  |  |  |  | l | Maceration | 1 |  |  |
|  |  |  |  | b | n | 1 |  |  |
|  |  |  |  | n |  | 1 |  |  |
| *^+^Garcinia kola* Heckel | 044246 | Clusiaceae | Ngadiadia (Kik.) | s | Masticate raw |  | 1 | 0,005 |
| *^+^Leonotis nepetifolia* (L.) R.Br. | 051606 | Lamiaceae | Manuansongi (Kik.) | l | Maceration |  | 2 | 0,009 |
| *^*^Mangifera indica* L. | 042871 | Anacardiaceae | Manga (Kik.) | b | Maceration | 1 | 2 | 0,009 |
|  |  |  |  |  | n | 1 |  |  |
| *^-^Manihot esculenta* Crantz | 042760 | Euphorbiaceae | Mandioca (Port.) | r | Raw | 3 | 9 | 0,041 |
|  |  |  |  |  | n | 6 |  |  |
| *^+^Maprounea africana* Müll.Arg. | 051640 | Euphorbiaceae | Nsiele nsiele (Kik.) | l | Raw | 1 | 3 | 0,014 |
|  |  |  |  |  | Masticate raw | 1 |  |  |
|  |  |  |  |  | n | 1 |  |  |
| *^+^Milicia excelsa* (Welw.) C.C.Berg | 051637 | Moraceae | Nkamba (Kik.), Moreira (Port.) | ss | Maceration | 3 | 8 | 0,036 |
|  |  |  |  |  | n | 5 |  |  |
| *^+^Momordica charantia* L. | 051646 | Cucurbitaceae | Dimbunzu, Lumbuzua, mbuzua, Mambuzu, (Kik.) | l | Maceration | 3 | 5 | 0,023 |
|  |  |  |  | p | 3 days maceration | 1 |  |  |
|  |  |  |  | n |  | 1 |  |  |
| *^+^Morinda lucida* Benth. | 051641 | Rubiaceae | Nsiki, masiki, nxiki (Kik.) | l | Infusion | 3 | 5 | 0,023 |
|  |  |  |  |  | infusion with Kongobololo (*Morinda morindoides*) leaves | 1 |  |  |
|  |  |  |  | r |  | 1 |  |  |
|  |  |  |  | n |  | 2 |  |  |
| *^+^Morinda morindoides* (Baker) Milne-Redh. | 051623 | Rubiaceae | Disu dia lunguenia, Meso-nkama (Kik.), Kongobololo, Nkongobololo (Kik.) | l | Infusion | 6 | 10 | 0,045 |
|  |  |  |  |  | Infusion with Masiki (*Morinda lucida*) leaves | 1 |  |  |
|  |  |  |  | n |  | 3 |  |  |
| *^+^Periploca nigrescens* Afzel. | 053481 | Apocynaceae | Lombua, Malombua (Kik.) | n |  | 1 | 1 | 0,005 |
| *^+^Psorospermum febrifugum* Spach | 051609 | Hypericaceae | Fiofio (Kik.) | l | Masticate raw | 1 | 1 | 0,005 |
| ^+^*Sesamum indicum* L. | 043895 | Pedaliaceae | Gergelim (Port.), Wanguila (Kik.) | n |  |  | 2 | 0,009 |
| *^+^Sesbania sphaerosperma* Welw. | 051652 | Fabaceae | Minzenze, Munzenze (Kik.) | n |  |  | 1 | 0,005 |
| *^*^Spondias mombin* L. | 051647 | Anacardiaceae | Gajajeira, Gajaja (Port.), Mungiengie (Kik.), Mingiengie (Kik.) | l | Masticate raw | 4 | 80 | 0,364 |
|  |  |  |  |  | Maceration | 7 |  |  |
|  |  |  |  |  | Infusion | 6 |  |  |
|  |  |  |  |  | Decoction | 5 |  |  |
|  |  |  |  |  | Raw | 4 |  |  |
|  |  |  |  |  | n | 20 |  |  |
|  |  |  |  | b | Infusion | 1 |  |  |
|  |  |  |  |  | 3 days Decoction | 1 |  |  |
|  |  |  |  |  | Maceration | 1 |  |  |
|  |  |  |  |  | n | 2 |  |  |
|  |  |  |  | n |  | 32 |  |  |
| *^+^**Syzygium guineense* (Willd.) DC. | 051632 | Myrtaceae | Monguacuma (Kik.) | b | Decoction and drunken with Mpeve (*Monodora myristica*) and Nkuakua (*Xylopia aethiopica*) | 1 | 2 | 0,009 |
|  |  |  |  |  | Raw | 1 |  |  |
|  |  |  |  |  | With Ginguba (*Arachis hypogaea*) | 1 |  |  |
| *^+^Tetracera poggei* Gilg | 053477 | Dilleniaceae | Nsingu nkayi, Nsingu a nkayi (Kik.) | l | Infusion | 1 | 2 | 0,009 |
|  |  |  |  | n |  | 1 |  |  |
| *^+^Vernonia amygdalina* Delile | 051610 | Asteraceae | Malulu, Malulua (Kik.) | l | Maceration | 7 | 24 | 0,109 |
|  |  |  |  |  | Infusion | 1 |  |  |
|  |  |  |  |  | Masticate raw | 1 |  |  |
|  |  |  |  |  | n | 2 |  |  |
|  |  |  |  | n | Maceration | 1 |  |  |
|  |  |  |  |  | n | 12 |  |  |
| *Vitex* spec*.*^•^ |  | Lamiaceae | Mafilu (Kik.) | n |  |  | 1 | 0,005 |
| Bitter plants |  |  |  |  |  |  | 1 | 0,005 |
|  |  |  | Kimvumina (Kik.) | l |  | 1 | 2 | 0,009 |
|  |  |  |  | n |  | 1 |  |  |
|  |  |  | Kunsevi (Kik.) | n |  |  | 1 | 0,005 |
|  |  |  | Manzenze (Kik.) | n |  |  | 1 | 0,005 |

Table 7: Overview of plants, foods and treatments a lactating mother should not use. All identified plant species were categorised in endemic (E), naturalised (*), listed (+) and not listed (-) (Neuwinger 2000). For most plants, the herbarium number according to Herbarium Dresdense (HD) is listed. In cases where no herbarium specimen was collected the number of a photo proof (F or G-F) is given. Plants, which were identified with the comparison of the Portuguese or Kikongo names with the data collection of Lautenschläger (2018)19 were marked with an •; part: b = bark, ba = bacon, bo = bone, cl = clay, f = fruit, l = leaves, o = oil, p = whole plant, r = root, s= seed, ss = stem sap, st = stem; n = no further information; Total number of citations in the interviews (CI), number of interviews (N), KiKongo name (Kik), Portuguese name (Port.)

| **Scientific name** | **Herbarium Number** | **Family** | **Local name** | **Part** | **Preparation** | **Citation** | **CI** | **RFC** (N = 236) | **Additional information** |
| --- | --- | --- | --- | --- | --- | --- | --- | --- | --- |
| *^+^Abelmoschus esculentus* (L.) Moench | G5b  051611 | Malvaceae | Quiabo (Port.) | n |  |  | 1 | 0,004 | Milk becomes sour |
| *^?^Aframomum melegueta* K.Schum^•^ | 044226 | Zingiberaceae | Ndungu za kongo (Kik.) | n |  |  | 1 | 0,004 |  |
| *^*^Amaranthus caudatus* L. | 051616 | Amaranthaceae | Gimboa (Kik.) | l |  | 1 | 3 | 0,013 | Milk becomes watery |
|  |  |  |  | n |  | 2 |  |  |  |
| *^*^Arachis hypogaea* L. |  | Fabaceae | Ginguba (Kik.) | s | dry | 1 | 2 | 0,008 |  |
|  |  |  |  | n |  | 1 |  |  |  |
| *^-^Capsicum annuum* L.^•^ | 042694 | Solanaceae | Ndungu, Ndungu za matebo (Kik.) |  | With salt |  | 3 | 0,013 |  |
|  |  |  |  |  | n |  |  |  |  |
| *^-^Citrus reticulata* Blanco | 051621 | Rutaceae | Tangerina (Port.) | f |  | 1 | 2 | 0,008 |  |
|  |  |  |  | n |  | 1 |  |  |  |
| *Citrus* spec. |  | Rutaceae | Laranja (Port.) |  |  |  | 1 | 0,004 |  |
| *^+^Crassocephalum rubens* (Juss. ex Jacq.) S.Moore | 051645 | Asteraceae | Bungudia (Kik.) | l |  | 2 | 3 | 0,013 |  |
|  |  |  |  | p |  | 1 |  |  |  |
|  |  |  |  | n |  | 1 |  |  |  |
| *Cucurbita* spec. |  | Cucurbitaceae | Muedi, Muteta (Kik.), Abóbora (Port.) | l |  | 2 | 9 | 0,038 | Milk becomes watery and child suffers from diarrhoea |
|  |  |  |  | f |  | 1 |  |  |  |
|  |  |  |  | n |  | 6 |  |  |  |
| *^+^Dacryodes edulis* (G.Don) H.J.Lam | F_37  G-F7 | Burseaceae | N´safu (Kik.) |  |  |  | 1 | 0,004 | Child suffers from diarrhoea |
| *^+^Elaeis guineensis* Jacq. | G-F3  G-F4  G-F5 | Arecaceae | Dendê (Port.) | f |  | 4 | 10 | 0,042 | Milk turns yellow and child suffers from diarrhoea and stomach pains |
|  |  |  |  | o |  | 4 |  |  |  |
|  |  |  |  | n |  | 2 |  |  |  |
| *^+^Garcinia kola* Heckel | 044246 | Clusiaceae | Ngadiadia (Kik.) | n |  |  | 1 | 0,004 | Milk becomes watery |
| *Hibiscus* spec. | 051650 | Malvaceae | Kixixi (Kik.) | n |  |  | 1 | 0,004 |  |
| *^*^Mangifera indica* L. | 042871 | Anacardiaceae | Manga (Port.) | f |  | 6 | 17 | 0,072 | Milk becomes watery |
|  |  |  |  | n |  | 11 |  |  |  |
| *^-^Manihot esculenta* Crantz | 042760 | Euphorbiaceae | Kisaka (Kik.), Mandioca (Port.) | l |  | 1 | 7 | 0,030 | No consumption of Funge for 3 months after birth; if the mother eats the leaves milk gets watery and child suffers from diarrhoea |
|  |  |  |  | r | mash | 6 |  |  |  |
| *^+^Maprounea africana* Müll.Arg. | 051640 | Euphorbiaceae | Nsiele nsiele (Kik.) | n |  |  | 1 | 0,004 |  |
| *^+^Milicia excelsa* (Welw.) C.C.Berg | 051637 | Moraceae | Nkamba (Kik.), Moreira (Port.) | ss |  |  | 1 | 0,004 |  |
| *^+^Monodora myristica* (Gaertn.) Dunal | 044707 | Annonaceae | Mpeve (Kik.) | f |  | 1 | 6 | 0,025 | Milk dries up |
|  |  |  |  | n |  | 5 |  |  |  |
| *^+^Morinda morindoides* (Baker) Milne-Redh. | 051623 | Rubiaceae | Disu dia lunguenia, Meso-nkama, Kongobololo, Nkongobololo (Kik.) | n |  |  | 1 | 0,004 | Don't use during pregnancy |
| *Musa* spec. |  | Musaceae | Banana (Port.) |  |  |  | 1 | 0,004 | Milk becomes watery |
| *^-^Oryza sativa* L. |  | Poaceae | Arroz (Port.) |  |  |  | 1 | 0,004 |  |
| *^*^Phaseolus vulgaris* L.^•^ | 042758 | Fabaceae | Feijões (Port.) | s |  |  | 1 | 0,004 |  |
| *^+^Piper guineense* Schumach. & Thonn. | 051633 | Piperaceae | Kapidi, Kupidi (Kik.) | s |  | 1 | 2 | 0,008 |  |
|  |  |  |  | n |  | 1 |  |  |  |
| *^+^Pteridium centrali-africanum* (Hieron.) Alston | 051644 | Dennstaedtiaceae | Feto (Kik.) | st | decoction |  | 1 | 0,004 |  |
| *Rumex abyssinicus* Jacq. | 053478 |  | Sengan nzukula (Kik.) | l |  |  | 1 | 0,004 |  |
| *^*^Saccharum officinarum* L*.* |  | Poaceae | Cana de açúcar (Port.) | st |  |  | 3 | 0,013 | Milk becomes watery |
| *^+^Salacia erecta* (G.Don) Walp. | 053479  053480 | Celastraceae | Mbonda, Kanzangu (Kik.) | l | infusion | 2 | 12 | 0,051 | Milk drys up |
|  |  |  |  |  | decoction | 1 |  |  |  |
|  |  |  |  |  | n | 3 |  |  |  |
|  |  |  |  | n |  | 6 |  |  |  |
| *^*^Spondias mombin* L. | 051647 | Anacardiaceae | Mungiengie (Kik.), Mingiengie (Kik.),  Gajajeira (Port.) | l |  |  | 1 | 0,004 | Milk becomes watery and child suffers from diarrhoea |
| *^+^Vernonia amygdalina* Delile | 051610 | Asteraceae | Malulu, Malulua (Kik.) | l |  | 3 | 3 | 0,013 | Milk becomes watery and child suffers from diarrhoea |
|  |  |  |  | p |  | 1 |  |  |  |
| Patterned antelope |  |  |  | m |  |  | 1 | 0,004 |  |
| Bird |  |  | Pássaro (Port.) | m |  |  | 6 | 0,025 |  |
| Bird's nest |  |  | Ninho de pássaros (Port.) |  | Take away |  | 2 | 0,008 |  |
| Bitter plants |  |  |  |  |  |  | 1 | 0,004 |  |
| Chicken |  |  | Galinha (Port.) | m |  |  | 1 | 0,004 |  |
| Eggs |  |  | Ovos (Port.) |  |  |  | 1 | 0,004 | Child becomes sick |
| Fish |  |  | Peixe (Port.) |  | Dried | 37 | 38 | 0,161 | Milk becomes watery and child suffers from diarrhoea |
|  |  |  |  |  | n | 1 |  |  |  |
| Goat |  |  | Cabra (Port.) | m |  |  | 10 | 0,042 | Child becomes scrabies; child dies |
| Meat with vegetables |  |  |  |  |  |  | 1 | 0,004 |  |
| Pork |  |  | Porco (Port.) | m |  |  | 1 | 0,004 |  |
| Salt |  |  | Sal (Port.) |  |  |  | 1 | 0,004 |  |
|  |  |  | Casca (Port.) |  |  |  | 1 | 0,004 | Don't use during pregnancy |
|  |  |  | Mabele (Kik.) | n |  |  | 1 | 0,004 |  |
|  |  |  | Nganzi (Kik.) | n |  |  | 1 | 0,004 | Don't use during pregnancy |

Table 8: Overview of plants, foods and treatments, which are used to decrease the breast milk production. All identified plant species were categorised in endemic (E), naturalised (*), listed (+) and not listed (-) (Neuwinger 2000). For most plants, the herbarium number according to Herbarium Dresdense (HD) is listed. In cases where no herbarium specimen was collected the number of a photo proof (F or G-F) is given. Plants which were identified with the comparison of the Portuguese or Kikongo names with the data collection of Lautenschläger (2018)19 were marked with an •; part: b = bark, ba = bacon, bo = bone, cl = clay, f = fruit, l = leaves, o = oil, p = whole plant, r = root, s= seed, ss = stem sap, st = stem; n = no further information; Total number of citations in the interviews (CI), number of interviews (N), KiKongo name (Kik), Portuguese name (Port.)

| **Scientific name** | **Herbarium Number** | **Family** | **Local name** | **Part** | **Preparation** | **Citations** | **CI** | **RFC** (N = 124) |
| --- | --- | --- | --- | --- | --- | --- | --- | --- |
| *^+^Abrus precatorius* L. | 051649 | Fabaceae | Kiambiembie (Kik.) | l | Maceration | 1 | 2 | 0,016 |
|  |  |  |  | n |  | 1 |  |  |
| *^-^Canavalia gladiata* (Jacq.) DC. | F_19 | Fabaceae | Nkasa (Kik.), Feijão (Port.) | s | Maceration with Ntowani (*Phyllanthus* spec.) leaves and Ngidingonda(a specific stone) |  | 1 | 0,008 |
| *Costus* spec. | 051608 | Costaceae | Nsangalavua (Kik.) | n |  |  | 1 | 0,008 |
| *^+^Crassocephalum rubens* (Juss. ex Jacq.) S.Moore | 051645 | Asteraceae | Bungudia (Kik.) | n |  |  | 1 | 0,008 |
| *Cucurbita* spec. |  | Cucurbitaceae | Muteta, Abóbora (Port.), Muengeleka (Kik.) | n |  |  | 1 | 0,008 |
| *^+^Elaeis guineensis* Jacq. | G-F3  G-F4  G-F5 | Arecaceae | Kokonote (Kik.) | s | With Mandioca (*Manihot esculenta*) and cana de açúcar (*Saccharum officinarum*) |  | 1 | 0,008 |
| *^+^Leonotis nepetifolia* (L.) R.Br. | 051606 | Lamiaceae | Manuansongi (Kik.) | l | Maceration |  | 1 | 0,008 |
| *^-^Manihot esculenta* Crantz | 042760 | Euphorbiaceae | Mandioca (Port.) | r | With Kokonote (*Elaeis guineensis*) and Cana de açúcar (*Saccharum officinarum*) |  | 1 | 0,008 |
| *^+^Maprounea africana* Müll.Arg. | 051640 | Euphorbiaceae | Nsele nsiele (Kik.) | l | Maceration |  | 1 | 0,008 |
| *^+^Milicia excelsa* (Welw.) C.C.Berg | 051637 | Moraceae | Nkamba (Kik.), Moreira (Port.) | ss |  |  | 1 | 0,008 |
| *^+^Monodora myristica* (Gaertn.) Dunal | 044707 | Annonaceae | Mpeve (Kik.) | s | Masticate raw and apply on the breast | 1 | 3 | 0,024 |
|  |  |  |  | n | Masticate raw | 1 |  |  |
|  |  |  |  |  | n | 1 |  |  |
| *Phyllanthus* spec. | 051651 | Phyllanthaceae | Ntowani (Kik.) | l | Maceration with Nkasa (*Canavalia gladiata*) seeds and Ngidingonda (a specific stone) |  | 1 | 0,008 |
| *^*^Saccharum officinarum* L*.* |  | Poaceae | Cana de açúcar (Port.) | st | With Kokonote (*Elaeis guineensis*) and Mandioca (*Manihot esculenta*) | 1 | 2 | 0,016 |
|  |  |  |  |  | n | 1 |  |  |
| ^+^*Salacia erecta* (G.Don) Walp. | 053479  053480 | Celastraceae | Mbonda (Kik.) | l | Infusion | 1 | 2 | 0,016 |
|  |  |  |  |  | n | 1 |  |  |
| *^+^Sesamum indicum L.* | 043895 | Pedaliaceae | Gergelim (Port.), Wanguila (Kik.) | S |  |  | 1 | 0,008 |
| *^+^Vernonia amygdalina* Delile | 051619 | Asteraceae | Malulu, Malulua (Kik.) | l | Infusion |  | 1 | 0,008 |
| *^+^Xylopia aethiopica* (Dunal) A.Rich. | F_71  044264 | Annonaceae | Nkuwa nkuwa, Nkuakua (Kik.) | n |  |  | 1 | 0,008 |
| Clean the body |  |  |  |  |  |  | 2 | 0,016 |
| Express breast milk |  |  |  |  |  |  | 1 | 0,008 |
| Fish |  |  | Peixe (Port.) |  | Dried |  | 1 | 0,008 |
| massage |  |  |  |  |  |  | 1 | 0,008 |
| Reduced food intake |  |  |  |  |  |  | 2 | 0,016 |
|  |  |  | Bulukutu (Kik.) |  | Infusion |  | 1 | 0,008 |
|  |  |  | Kangudi (Kik.) |  |  |  | 1 | 0,008 |
|  |  |  | Ngidingonda (Kik.) | A specific kind of stone | Maceration with Ntowani (*Phyllanthus* spec.) leaves and Nkasa (*Canavalia gladiata*) seeds |  | 1 | 0,008 |
