# Supplementary material for: Plants, food and treatments used by BaKongo tribes in Uíge (northern Angola) to affect the quality and quantity of human breast milk
Source: Int Breastfeed J. 2020 Oct 23;15:88. doi: 10.1186/s13006-020-00329-1 (PMC7583195; doi:10.1186/s13006-020-00329-1)
Supplement: Supplementary file 2 — Additional file 2:. Locations of the villages where the interviews were conducted. [file 13006_2020_329_MOESM2_ESM.docx]

Table 5: Overview of plants, foods and treatments which increase or promote lactation. All identified plant species were categorised in endemic (E), naturalised (*), listed (+) and not listed (-) (Neuwinger 2000). For most plants the herbarium number according to Herbarium Dresdense (DR) is listed. In cases where no herbarium specimen was collected the number of a photo proof (F or G-F) is given. Plants which were identified with the comparison of the Portuguese or Kikongo names with the data collection of Lautenschläger (2018)^19^ were marked with an •. To verify the use as a galactagogue the following references were checked: 1 = Neuwinger (2000); 2 = Iwu (2014); 3 = Latham (2014); 4 = Konda ku Mbuta (2012); 5 = Kokwardo (2009). If the literature states another use than as a galactagogue it is shown by a “- “. A ”/” points out that no information about this plant is given. Only plants used as a galactagogue as recorded are marked with a “+”. If the plant is used as a galactagogue but there is another part of the plant or another preparation cart in the literature this is shown by an “o”. part: b = bark, ba = bacon, bo = bone, c = coconut milk, cl = clay, f = fruit, l = leaves, r = root, s= seed, ss = stem sap, st = stem; n = no further information; Total number of citations in the interviews (CI), number of interviews (N), KiKongo name (Kik), Portuguese name (Port.)

| **Scientific name** | **Herbarium number DR** | **Family** | **Local name** | **Part** | **Preparation** | **Citations** | **CI** | **RFC** (N = 259) | **References** |
| --- | --- | --- | --- | --- | --- | --- | --- | --- | --- |
| *^+^Abrus precatorius* L. | 051649 | Fabaceae | Maique, Kiambiembie (Kik.) | r |  | 1 | 3 | 0,012 | -^1^; -^2^; +^3^; o^4^; -^5^; |
|  |  |  |  | l |  | 2 |  |  |  |
| *^+^Aframomum alboviolaceum* (Ridl.) K. Schum. | 051614 | Zingiberaceae | Gingenga da queimada (Port.) | f |  | 1 | 1 | 0,004 | -^1^; /^2^; -^3^; /^4^; /^5^; |
| *^*^Amaranthus caudatus* L. | 051616 | Amaranthaceae | Gimboa, Bowa (Kik.) | l |  | 2 | 4 | 0,012 | -^1^; -^2^; /^3^; -^4^; -^5^; |
|  |  |  |  | st |  | 1 |  |  |  |
|  |  |  |  | n |  | 1 |  |  |  |
| *^*^Arachis hypogaea* L. | G-F10 | Fabaceae | Ginguba (Kik.) | s | fresh | 54 | 95 | 0,367 | +^1^; -^2^; -^3^; -^4^; /^5^; |
|  |  |  |  |  | any form | 3 |  |  |  |
|  |  |  |  |  | With salt (dermal) | 1 |  |  |  |
|  |  |  |  |  | n | 37 |  |  |  |
| *Brassica* spec.^•^ | 042794 | Brassicaceae | Couve (Port.), Nkove (Kik.) | l | decoction | 2 | 10 | 0,039 | *Brassica integrifolia* (root) +^1^; *Brassica juncea* (leaves, oil) +^2^; -^3^; /^4^; *Brassica carinata* +^5^; |
|  |  |  |  |  | raw | 1 |  |  |  |
|  |  |  |  |  | n | 7 |  |  |  |
| *^+^Brillantaisia owariensis* P.Beauv. | 051631 | Acanthaceae | Lemba Lemba (Kik.) | l | infusion | 1 | 1 | 0,004 | -^1^; -^2^; -^3^; -^4^; /^5^; |
| *^*^Carica papaya* L. | F_22 | Caricaceae | Mamão (Port.), Papayi (Kik.) | f |  |  | 1 | 0,004 | -^1^; -^2^; -^3^; -^4^; /^5^; |
| *^*^Cocos nucifera* L. | G-F1  G-F2 | Arecaceae | Coco (Port.) | f | With Kokonote (*Elaeis guineensis*) | 1 | 3 | 0,012 | -^1^; -^2^; -^3^; /^4^; /^5^; |
|  |  |  |  | c |  | 2 |  |  |  |
| *^+^Combretum* *racemosum* P.Beauv. | 053487 | Combretaceae | Nsumbisila, Nsumbila (Kik.) | n |  |  | 1 | 0,004 | -^1^; -^2^; /^3^; -^4^; /^5^; |
| *^+^Costus afer* Ker Gawl. | 051612 | Costaceae | Mikeni (Kik.) | n |  |  | 1 | 0,004 | -^1^; -^2^; -^3^; -^4^; /^5^; |
| *Costus* spec. | 051608  053482 | Costaceae | Nsangalavua (Kik.) | st | With salt | 2 | 4 | 0,015 | *Costus lucanisianus* (twig sap) +^1^; *Costus lucanisianus* (leaves sap, aerial parts) +^2^; -^3^; -^4^; -^5^; |
|  |  |  |  |  | With Mandioca (*Manihot esculenta*) and Kokonote (*Elaeis guineensis*) |  |  |  |  |
|  |  |  |  | n |  | 2 |  |  |  |
| *Craterispermum* spec. | 053476 | Rubiaceae | Kiseka seka, Nseka-seka (Kik.) | r | Eat with raw Mandioca (*Manihot esculenta*) and Ginguba (*Arachis hypogaea*) | 1 | 3 | 0,012 | -^1^; -^2^; -^3^; -^4^; -^5^; |
|  |  |  |  | b | eat, chew | 2 |  |  |  |
| *Cucurbita* spec. |  | Cucurbitaceae | Muteta, Abóbora (Port.), Muengeleka (Kik.) | s | decoction | 1 | 6 | 0,023 | -^1^; -^2^; -^3^; /^4^; /^5^; |
|  |  |  |  |  | Grind and prepare like a small cake | 3 |  |  |  |
|  |  |  |  | l |  | 1 |  |  |  |
|  |  |  |  | n |  | 2 |  |  |  |
| *^+^Elaeis guineensis* Jacq. | G-F3  G-F4  G-F5 | Arecaceae | Kokonote (Kik.) | s | Raw | 1 | 17 | 0,066 | -^1^; o^2^; -^3^; -^4^; -^5^; |
|  |  |  |  |  | With Mandioca (*Manihot esculenta*) | 1 |  |  |  |
|  |  |  |  |  | With coconut | 1 |  |  |  |
|  |  |  |  |  | With Mandioca (*Manihot esculenta*) and Sangalavua (*Costus* spec.) | 1 |  |  |  |
|  |  |  |  |  | n | 13 |  |  |  |
| *^*^Euphorbia hirta* L. | 051624  051625 | Euphorbiaceae | Kimvumina, Kimvumina kia nkombo (Kik.) | l | maceration | 1 | 1 | 0,004 | o^1^; +^2^; +^3^; -^4^; o^5^; |
| *^+^Gongronema latifolium* Benth. | 051613 | Apocynaceae | Kimvumina (Kik.) | n |  |  | 4 | 0,015 | -^1^; -^2^; /^3^; /^4^; /^5^; |
| *^+^Heinsia crinita* (Afzel.) G.Taylor | 053486  051950 | Rubiaceae | Nsangumuni (Kik.) | n |  |  | 1 | 0,004 | -^1^; -^2^; -^3^; -^4^; -^5^; |
| *^*^Jatropha curcas* L. | 051620 | Euphorbiaceae | Mpuluka (Kik.) | l | Infusion | 1 | 2 | 0,008 | -^1^; o^2^; -^3^; -^4^; -^5^; |
|  |  |  |  |  | maceration | 1 |  |  |  |
| *^*^Mangifera indica* L. | 42871 | Anacardiaceae | Manga (Port.) | n |  |  | 1 | 0,004 | -^1^; -^2^; -^3^; -^4^; /^5^; |
| *^-^Manihot esculenta* Crantz | 42760 | Euphorbiaceae | Mandioca (Port.), Funge, Kisaka, N´saki (Kik.) | l | Raw | 1 | 147 | 0,568 | +^1^; /^2^; -^3^; -^4^; /^5^; |
|  |  |  |  |  | n | 45 |  |  |  |
|  |  |  |  | r | Raw | 56 |  |  |  |
|  |  |  |  |  | cooked | 1 |  |  |  |
|  |  |  |  |  | Row with Coconote (*Elaeis guineensis*) | 1 |  |  |  |
|  |  |  |  |  | n | 41 |  |  |  |
|  |  |  |  |  | Mash (Funge) | 2 |  |  |  |
| *^+^Maprounea africana* Müll.Arg. | 051640 | Euphorbiaceae | Nsiele nsiele (Kik.) | l | raw | 1 | 2 | 0,008 | -^1^; -^2^; /^3^; /^4^; -^5^; |
|  |  |  |  |  | n | 1 |  |  |  |
| *^+^Milicia excelsa* (Welw.) C.C.Berg | 051637 | Moraceae | Nkamba (Kik.), Moreira (Port.) | st | maceration | 1 | 3 | 0,012 | +^1^; +^2^; -^3^; -^4^; -^5^; |
|  |  |  |  | b | maceration | 1 |  |  |  |
|  |  |  |  | ss |  | 1 |  |  |  |
| *^+^Momordica charantia* L. | 051646 | Cucurbitaceae | Lumbuzua, Mambuzu (Kik.) | l | maceration | 1 | 2 | 0,008 | -^1^; -^2^; -^3^; /^4^; /^5^; |
|  |  |  |  | n |  | 1 |  |  |  |
| *Musa* spec. |  | Musaceae | Banana (Port.) | n |  |  | 1 | 0,004 | -^1^; -^2^; -^3^; -^4^; -^5^; |
| *^+^Periploca nigrescens* Afzel. | 053481 | Apocynaceae | Lombua, Malombua (Kik.) | b | Raw | 1 | 1 | 0,004 | -^1^; -^2^; /^3^; -^4^; /^5^; |
|  |  |  |  |  | maceration |  |  |  |  |
| *^-^Persea americana* Mill. |  | Lauraceae | Abacate (Port.) | l |  | 1 | 3 | 0,012 | -^1^; /^2^; -^3^; -^4^; /^5^; |
|  |  |  |  | n |  | 2 |  |  |  |
| *^*^Phaseolus vulgaris* L.^•^ | 42758 | Fabaceae | Feijões, (Port.)  Makasikila (Kik.) | s |  | 2 | 4 | 0,015 | /^1^; /^2^; -^3^; /^4^; /^5^; |
|  |  |  |  | l |  | 2 |  |  |  |
| *^+^Psophocarpus palustris* Desv. | 051638 | Fabaceae | Kikalakassa, Mpavu (Kik.) | l | Cooked and eaten with salt | 1 | 1 | 0,004 | -^1^; /^2^; / ^3^; /^4^; /^5^; |
| *^+^Psorospermum febrifugum* Spach | 051609 | Hypericaceae | Fiofio (Kik.) | l |  |  | 1 | 0,004 | -^1^; -^2^; -^3^; /^4^; -^5^; |
| *^+^Raphia matombe* De Wild. | 050857 | Arecaceae | Maruvo (Kik.) |  |  |  | 2 | 0,008 | /^1^; /^2^; -^3^; /^4^; /^5^; |
| *^*^Saccharum officinarum* L*.* |  | Poaceae | Cana de açúcar (Port.) | st |  |  | 12 | 0,046 | -^1^; -^2^; -^3^; /^4^; /^5^; |
| *^+^Sesamum indicum L.* | 043895 | Pedaliaceae | Gergelim (Port.), Wanguila (Kik.) | s | With Fungi | 1 | 31 | 0,120 | -^1^; o^2^; -^3^; /^4^; /^5^; |
|  |  |  |  |  | Raw and cooked | 1 |  |  |  |
|  |  |  |  |  | Cooked and eaten in food | 2 |  |  |  |
|  |  |  |  |  | n | 27 |  |  |  |
| *^+^Solanum macrocarpon* L.^•^ | 044099  G-F6 | Solanaceae | Couve preta (Port.) | l | decoction | 1 | 2 | 0,008 | -^1^; -^2^; -^3^; /^4^; /^5^; |
|  |  |  |  |  | n | 1 |  |  |  |
| *^*^Spondias mombin* L. | 051647 | Anacardiaceae | Mungiengie (Kik.), Mingiengie (Kik.),  Gajajeira (Port.) | l | masticate raw | 1 | 7 | 0,027 | -^1^; -^2^; -^3^; /^4^; /^5^; |
|  |  |  |  |  | infusion | 1 |  |  |  |
|  |  |  |  |  | n | 3 |  |  |  |
|  |  |  |  | r | n | 1 |  |  |  |
|  |  |  |  | n |  | 2 |  |  |  |
| *^+^Terminalia brachystemma* Welw. ex Hiern | 051607 | Combretaceae | Mungolo (Kik.) | r |  |  | 1 | 0,004 | -^1^; /^2^; /^3^; /^4^; /^5^; |
| *^+^Uvaria poggei* Engl. & Diels^•^ | 050910 | Annonaceae | Nkombo (Kik.) | n |  |  | 1 | 0,004 | -^1^; /^2^; /^3^; /^4^; /^5^; |
| *^+^Vernonia amygdalina* Delile | 051610 | Asteraceae | Malulu, Malulua (Kik.) | l | Maceration | 2 | 3 | 0,012 | -^1^; -^2^; -^3^; -^4^; -^5^; |
|  |  |  |  |  | masticate raw | 1 |  |  |  |
| *^+^Vitex doniana* Sweet | 053483 | Lamiaceae | Balafilo (Kik.) | n |  |  | 1 | 0,004 | +^1^; +^2^; -^3^; /^4^; -^5^; |
| *^*^Zea mays* L. |  | Poaceae | Milho (Port.) | s | Decoction with salt | 1 | 3 | 0,012 | -^1^; -^2^; -^3^; -^4^; /^5^; |
|  |  |  |  |  | Raw | 1 |  |  |  |
|  |  |  |  |  | n | 2 |  |  |  |
| Bird´s nest |  |  | Ninho de pássaros (Port.) |  | infusion |  | 1 | 0,004 |  |
| Eggs |  |  | Ovos (Port.) |  |  |  | 2 | 0,008 |  |
| Evaporated milk |  |  |  |  |  |  | 1 | 0,004 |  |
| Fish |  |  | Peixe, peixe seco (Port.) |  | Fresh | 1 | 18 | 0,069 |  |
|  |  |  |  |  | Dried | 17 |  |  |  |
| Meat |  |  |  |  |  |  | 1 | 0,004 |  |
| Milk |  |  | Vaca (Port.) |  |  |  | 2 | 0,008 |  |
| Minerals, Salt |  |  | Minérios, Sal (Port.) |  |  |  | 2 | 0,008 |  |
| Pork |  |  | Porco (Port.) | ba | n | 1 | 3 | 0,012 |  |
|  |  |  |  | bo | Decoction | 2 |  |  |  |
| Vegetables |  |  | Verduras (Port.) |  | Without meat | 1 | 2 | 0,008 |  |
|  |  |  |  |  | n | 1 |  |  |  |
| Mixture |  |  | milk, mgomagome (bark), Minkombo (root), Kapidi (*Piper guineense*), Mpeve (*Monodora myristica*), Nsaku (root), Manga (*Mangifera indica*) (bark) |  |  |  | 1 | 0,004 |  |
| mushroom |  |  | Utunturuo (Kik.), Kukumelo (Port.) |  |  |  | 1 | 0,004 | mushroom |
|  |  |  | Kimvumina, Kimvumina kia nkombo (Kik.) |  |  |  | 1 | 0,004 |  |
|  |  |  | Manquila, Maquila (Kik.) |  |  |  | 2 | 0,008 |  |
|  |  |  | Mungimba (Kik.) | r |  |  | 1 | 0,004 |  |
|  |  |  | Munzenzenzenze (Kik.) | l | Raw |  | 1 | 0,004 |  |
|  |  |  | Ngubanguba (Kik.) | l | infusion |  | 1 | 0,004 |  |
|  |  |  | Nkambiebie (Kik.) | l | maceration |  | 1 | 0,004 |  |
|  |  |  | Ntenda (Kik.) | r | With Mandioca (*Manihot esculenta*) | 1 | 2 | 0,008 |  |
|  |  |  |  | n |  | 1 |  |  |  |
|  | F8, F9, F10 |  | Mpemba, Luvemba, Mabele (Kik.) | cl | Decoction with a bone |  | 1 | 0,004 |  |
|  |  |  | Usa | l | Decoction and eaten with Ginguba (*Arachis hypogaea*) and salt |  | 1 | 0,004 |  |

Table 6: Overview of plants, foods and treatments, which are mentioned for “cleaning” the breast milk. All identified plant species were categorised in endemic (E), naturalised (*), listed (+) and not listed (-) (Neuwinger 2000). For most plants, the herbarium number according to Herbarium Dresdense (DR) is listed. In cases where no herbarium specimen was collected the number of a photo proof (F or G-F) is given. Plants, which were identified with the comparison of the Portuguese or Kikongo names with the data collection of Lautenschläger (2018) were marked with an •; part: b = bark, ba = bacon, bo = bone, cl = clay, f = fruit, l = leaves, o = oil, p = whole plant, r = root, s= seed, ss = stem sap, st = stem; n = no further information; Total number of citations in the interviews (CI), number of interviews (N), KiKongo name (Kik), Portuguese name (Port.)

| **Scientific name** | **Herbarium number DR** | **Family** | **Local name** | **Part** | **preparation** | **Citations** | **CI** | **RFC** (N = 220) |
| --- | --- | --- | --- | --- | --- | --- | --- | --- |
| *^+^Abrus precatorius* L. | 051649 | Fabaceae | Kiambiembie (Kik.) | l | Chewed | 6 | 9 | 0,041 |
|  |  |  |  |  | Maceration | 2 |  |  |
|  |  |  |  |  | n | 1 |  |  |
| *^+^Albizia adianthifolia* (Schum.) W.Wight | 051615 | Fabaceae | Mulu (Kik.) | n |  |  | 1 | 0,005 |
| *^*^Arachis hypogaea* L. |  | Fabaceae | Ginguba (Kik.) | s | Row | 1 | 2 | 0,009 |
|  |  |  |  |  | n | 1 |  |  |
| ^-^*Azadirachta indica* A.Juss. | 051636 | Meliaceae | Curatudo (Port.) | l | Maceration | 1 | 2 | 0,009 |
|  |  |  |  | n |  | 1 |  |  |
| *^+^Bauhinia thonningii* Schum.^•^ | 043847 | Fabaceae | Loloa (Kik.) | r | Maceration | 1 | 2 | 0,009 |
|  |  |  |  | l | Maceration | 1 |  |  |
| *^*^Carica papaya* L. | F_22 | Caricaceae | Mamão (Port.), Papayi (Kik.) | l | Maceration | 11 | 15 | 0,068 |
|  |  |  |  |  | n | 4 |  |  |
| *^+^Cayratia gracilis* (Guill. & Perr.) Suess. | 053475 | Vitaceae | Lembozi (Kik.) | l | Maceration | 1 | 2 | 0,009 |
|  |  |  |  | n |  | 1 |  |  |
| *^*^Cocos nucifera* L. | G-F1  G-F2 | Arecaceae | Coco (Port.) | f |  |  | 4 | 0,018 |
| *Craterispermum* spec. | 053485  053484 |  | Nseka seka (Kik.) | n |  |  | 1 | 0,005 |
| *^+^Dacryodes edulis* (G.Don) H.J.Lam | F_37  G-F7 | Burseaceae | N´safu (Kik.) | b | Maceration |  | 1 | 0,005 |
| *^-^Dysphania ambrosioides* (L.) Mosyakin & Clemants | 051634 | Amaranthaceae | Santa Maria (Port.) | n | With raw Mandioca (*Manihot esculenta*) |  | 1 | 0,005 |
| *^+^Elaeis guineensis* Jacq. | G-F3  G-F4  G-F5 | Arecaceae | Kokonote (Kik.) | s |  |  | 15 | 0,068 |
| *^+^Entada abyssinica* A.Rich. | 051627 | Fabaceae | Nsofi (Kik.) | l | Raw |  | 1 | 0,005 |
| *^*^Euphorbia hirta* L. | 051624  051625 | Euphorbiaceae | Kimvumina, Kimvumina kia nkombo (Kik.) | l | With Kokonote (*Elaeis guineensis*) | 1 | 1 | 0,005 |
| *^+^Gongronema latifolium* Benth. | 051613 | Apocynaceae | Kimvumina (Kik.) | st | Maceration | 2 | 5 | 0,023 |
|  |  |  |  | l | Maceration | 1 |  |  |
|  |  |  |  | b | n | 1 |  |  |
|  |  |  |  | n |  | 1 |  |  |
| *^+^Garcinia kola* Heckel | 044246 | Clusiaceae | Ngadiadia (Kik.) | s | Masticate raw |  | 1 | 0,005 |
| *^+^Leonotis nepetifolia* (L.) R.Br. | 051606 | Lamiaceae | Manuansongi (Kik.) | l | Maceration |  | 2 | 0,009 |
| *^*^Mangifera indica* L. | 042871 | Anacardiaceae | Manga (Kik.) | b | Maceration | 1 | 2 | 0,009 |
|  |  |  |  |  | n | 1 |  |  |
| *^-^Manihot esculenta* Crantz | 042760 | Euphorbiaceae | Mandioca (Port.) | r | Raw | 3 | 9 | 0,041 |
|  |  |  |  |  | n | 6 |  |  |
| *^+^Maprounea africana* Müll.Arg. | 051640 | Euphorbiaceae | Nsiele nsiele (Kik.) | l | Raw | 1 | 3 | 0,014 |
|  |  |  |  |  | Masticate raw | 1 |  |  |
|  |  |  |  |  | n | 1 |  |  |
| *^+^Milicia excelsa* (Welw.) C.C.Berg | 051637 | Moraceae | Nkamba (Kik.), Moreira (Port.) | ss | Maceration | 3 | 8 | 0,036 |
|  |  |  |  |  | n | 5 |  |  |
| *^+^Momordica charantia* L. | 051646 | Cucurbitaceae | Dimbunzu, Lumbuzua, mbuzua, Mambuzu, (Kik.) | l | Maceration | 3 | 5 | 0,023 |
|  |  |  |  | p | 3 days maceration | 1 |  |  |
|  |  |  |  | n |  | 1 |  |  |
| *^+^Morinda lucida* Benth. | 051641 | Rubiaceae | Nsiki, masiki, nxiki (Kik.) | l | Infusion | 3 | 5 | 0,023 |
|  |  |  |  |  | infusion with Kongobololo (*Morinda morindoides*) leaves | 1 |  |  |
|  |  |  |  | r |  | 1 |  |  |
|  |  |  |  | n |  | 2 |  |  |
| *^+^Morinda morindoides* (Baker) Milne-Redh. | 051623 | Rubiaceae | Disu dia lunguenia, Meso-nkama (Kik.), Kongobololo, Nkongobololo (Kik.) | l | Infusion | 6 | 10 | 0,045 |
|  |  |  |  |  | Infusion with Masiki (*Morinda lucida*) leaves | 1 |  |  |
|  |  |  |  | n |  | 3 |  |  |
| *^+^Periploca nigrescens* Afzel. | 053481 | Apocynaceae | Lombua, Malombua (Kik.) | n |  | 1 | 1 | 0,005 |
| *^+^Psorospermum febrifugum* Spach | 051609 | Hypericaceae | Fiofio (Kik.) | l | Masticate raw | 1 | 1 | 0,005 |
| ^+^*Sesamum indicum* L. | 043895 | Pedaliaceae | Gergelim (Port.), Wanguila (Kik.) | n |  |  | 2 | 0,009 |
| *^+^Sesbania sphaerosperma* Welw. | 051652 | Fabaceae | Minzenze, Munzenze (Kik.) | n |  |  | 1 | 0,005 |
| *^*^Spondias mombin* L. | 051647 | Anacardiaceae | Gajajeira, Gajaja (Port.), Mungiengie (Kik.), Mingiengie (Kik.) | l | Masticate raw | 4 | 80 | 0,364 |
|  |  |  |  |  | Maceration | 7 |  |  |
|  |  |  |  |  | Infusion | 6 |  |  |
|  |  |  |  |  | Decoction | 5 |  |  |
|  |  |  |  |  | Raw | 4 |  |  |
|  |  |  |  |  | n | 20 |  |  |
|  |  |  |  | b | Infusion | 1 |  |  |
|  |  |  |  |  | 3 days Decoction | 1 |  |  |
|  |  |  |  |  | Maceration | 1 |  |  |
|  |  |  |  |  | n | 2 |  |  |
|  |  |  |  | n |  | 32 |  |  |
| *^+^**Syzygium guineense* (Willd.) DC. | 051632 | Myrtaceae | Monguacuma (Kik.) | b | Decoction and drunken with Mpeve (*Monodora myristica*) and Nkuakua (*Xylopia aethiopica*) | 1 | 2 | 0,009 |
|  |  |  |  |  | Raw | 1 |  |  |
|  |  |  |  |  | With Ginguba (*Arachis hypogaea*) | 1 |  |  |
| *^+^Tetracera poggei* Gilg | 053477 | Dilleniaceae | Nsingu nkayi, Nsingu a nkayi (Kik.) | l | Infusion | 1 | 2 | 0,009 |
|  |  |  |  | n |  | 1 |  |  |
| *^+^Vernonia amygdalina* Delile | 051610 | Asteraceae | Malulu, Malulua (Kik.) | l | Maceration | 7 | 24 | 0,109 |
|  |  |  |  |  | Infusion | 1 |  |  |
|  |  |  |  |  | Masticate raw | 1 |  |  |
|  |  |  |  |  | n | 2 |  |  |
|  |  |  |  | n | Maceration | 1 |  |  |
|  |  |  |  |  | n | 12 |  |  |
| *Vitex* spec*.*^•^ |  | Lamiaceae | Mafilu (Kik.) | n |  |  | 1 | 0,005 |
| Bitter plants |  |  |  |  |  |  | 1 | 0,005 |
|  |  |  | Kimvumina (Kik.) | l |  | 1 | 2 | 0,009 |
|  |  |  |  | n |  | 1 |  |  |
|  |  |  | Kunsevi (Kik.) | n |  |  | 1 | 0,005 |
|  |  |  | Manzenze (Kik.) | n |  |  | 1 | 0,005 |

Table 7: Overview of plants, foods and treatments a lactating mother should not use. All identified plant species were categorised in endemic (E), naturalised (*), listed (+) and not listed (-) (Neuwinger 2000). For most plants, the herbarium number according to Herbarium Dresdense (DR) is listed. In cases where no herbarium specimen was collected the number of a photo proof (F or G-F) is given. Plants, which were identified with the comparison of the Portuguese or Kikongo names with the data collection of Lautenschläger (2018)19 were marked with an •; part: b = bark, ba = bacon, bo = bone, cl = clay, f = fruit, l = leaves, o = oil, p = whole plant, r = root, s= seed, ss = stem sap, st = stem; n = no further information; Total number of citations in the interviews (CI), number of interviews (N), KiKongo name (Kik), Portuguese name (Port.)

| **Scientific name** | **Herbarium Number DR** | **Family** | **Local name** | **Part** | **Preparation** | **Citation** | **CI** | **RFC** (N = 236) | **Additional information** |
| --- | --- | --- | --- | --- | --- | --- | --- | --- | --- |
| *^+^Abelmoschus esculentus* (L.) Moench | G-5b  051611 | Malvaceae | Quiabo (Port.) | n |  |  | 1 | 0,004 | Milk becomes sour |
| *^?^Aframomum melegueta* K.Schum^•^ | 044226 | Zingiberaceae | Ndungu za kongo (Kik.) | n |  |  | 1 | 0,004 |  |
| *^*^Amaranthus caudatus* L. | 051616 | Amaranthaceae | Gimboa (Kik.) | l |  | 1 | 3 | 0,013 | Milk becomes watery |
|  |  |  |  | n |  | 2 |  |  |  |
| *^*^Arachis hypogaea* L. |  | Fabaceae | Ginguba (Kik.) | s | dry | 1 | 2 | 0,008 |  |
|  |  |  |  | n |  | 1 |  |  |  |
| *^-^Capsicum annuum* L.^•^ | 042694 | Solanaceae | Ndungu, Ndungu za matebo (Kik.) |  | With salt |  | 3 | 0,013 |  |
|  |  |  |  |  | n |  |  |  |  |
| *^-^Citrus reticulata* Blanco | 051621 | Rutaceae | Tangerina (Port.) | f |  | 1 | 2 | 0,008 |  |
|  |  |  |  | n |  | 1 |  |  |  |
| *Citrus* spec. |  | Rutaceae | Laranja (Port.) |  |  |  | 1 | 0,004 |  |
| *^+^Crassocephalum rubens* (Juss. ex Jacq.) S.Moore | 051645 | Asteraceae | Bungudia (Kik.) | l |  | 2 | 3 | 0,013 |  |
|  |  |  |  | p |  | 1 |  |  |  |
|  |  |  |  | n |  | 1 |  |  |  |
| *Cucurbita* spec. |  | Cucurbitaceae | Muedi, Muteta (Kik.), Abóbora (Port.) | l |  | 2 | 9 | 0,038 | Milk becomes watery and child suffers from diarrhoea |
|  |  |  |  | f |  | 1 |  |  |  |
|  |  |  |  | n |  | 6 |  |  |  |
| *^+^Dacryodes edulis* (G.Don) H.J.Lam | F_37  G-F7 | Burseaceae | N´safu (Kik.) |  |  |  | 1 | 0,004 | Child suffers from diarrhoea |
| *^+^Elaeis guineensis* Jacq. | G-F3  G-F4  G-F5 | Arecaceae | Dendê (Port.) | f |  | 4 | 10 | 0,042 | Milk turns yellow and child suffers from diarrhoea and stomach pains |
|  |  |  |  | o |  | 4 |  |  |  |
|  |  |  |  | n |  | 2 |  |  |  |
| *^+^Garcinia kola* Heckel | 044246 | Clusiaceae | Ngadiadia (Kik.) | n |  |  | 1 | 0,004 | Milk becomes watery |
| *Hibiscus* spec. | 051650 | Malvaceae | Kixixi (Kik.) | n |  |  | 1 | 0,004 |  |
| *^*^Mangifera indica* L. | 042871 | Anacardiaceae | Manga (Port.) | f |  | 6 | 17 | 0,072 | Milk becomes watery |
|  |  |  |  | n |  | 11 |  |  |  |
| *^-^Manihot esculenta* Crantz | 042760 | Euphorbiaceae | Kisaka (Kik.), Mandioca (Port.) | l |  | 1 | 7 | 0,030 | No consumption of Funge for 3 months after birth; if the mother eats the leaves milk gets watery and child suffers from diarrhoea |
|  |  |  |  | r | mash | 6 |  |  |  |
| *^+^Maprounea africana* Müll.Arg. | 051640 | Euphorbiaceae | Nsiele nsiele (Kik.) | n |  |  | 1 | 0,004 |  |
| *^+^Milicia excelsa* (Welw.) C.C.Berg | 051637 | Moraceae | Nkamba (Kik.), Moreira (Port.) | ss |  |  | 1 | 0,004 |  |
| *^+^Monodora myristica* (Gaertn.) Dunal | 044707 | Annonaceae | Mpeve (Kik.) | f |  | 1 | 6 | 0,025 | Milk dries up |
|  |  |  |  | n |  | 5 |  |  |  |
| *^+^Morinda morindoides* (Baker) Milne-Redh. | 051623 | Rubiaceae | Disu dia lunguenia, Meso-nkama, Kongobololo, Nkongobololo (Kik.) | n |  |  | 1 | 0,004 | Don't use during pregnancy |
| *Musa* spec. |  | Musaceae | Banana (Port.) |  |  |  | 1 | 0,004 | Milk becomes watery |
| *^-^Oryza sativa* L. |  | Poaceae | Arroz (Port.) |  |  |  | 1 | 0,004 |  |
| *^*^Phaseolus vulgaris* L.^•^ | 042758 | Fabaceae | Feijões (Port.) | s |  |  | 1 | 0,004 |  |
| *^+^Piper guineense* Schumach. & Thonn. | 051633 | Piperaceae | Kapidi, Kupidi (Kik.) | s |  | 1 | 2 | 0,008 |  |
|  |  |  |  | n |  | 1 |  |  |  |
| *^+^Pteridium centrali-africanum* (Hieron.) Alston | 051644 | Dennstaedtiaceae | Feto (Kik.) | st | decoction |  | 1 | 0,004 |  |
| *Rumex abyssinicus* Jacq. | 053478 |  | Sengan nzukula (Kik.) | l |  |  | 1 | 0,004 |  |
| *^*^Saccharum officinarum* L*.* |  | Poaceae | Cana de açúcar (Port.) | st |  |  | 3 | 0,013 | Milk becomes watery |
| *^+^Salacia erecta* (G.Don) Walp. | 053479  053480 | Celastraceae | Mbonda, Kanzangu (Kik.) | l | infusion | 2 | 12 | 0,051 | Milk drys up |
|  |  |  |  |  | decoction | 1 |  |  |  |
|  |  |  |  |  | n | 3 |  |  |  |
|  |  |  |  | n |  | 6 |  |  |  |
| *^*^Spondias mombin* L. | 051647 | Anacardiaceae | Mungiengie (Kik.), Mingiengie (Kik.),  Gajajeira (Port.) | l |  |  | 1 | 0,004 | Milk becomes watery and child suffers from diarrhoea |
| *^+^Vernonia amygdalina* Delile | 051610 | Asteraceae | Malulu, Malulua (Kik.) | l |  | 3 | 3 | 0,013 | Milk becomes watery and child suffers from diarrhoea |
|  |  |  |  | p |  | 1 |  |  |  |
| Patterned antelope |  |  |  | m |  |  | 1 | 0,004 |  |
| Bird |  |  | Pássaro (Port.) | m |  |  | 6 | 0,025 |  |
| Bird's nest |  |  | Ninho de pássaros (Port.) |  | Take away |  | 2 | 0,008 |  |
| Bitter plants |  |  |  |  |  |  | 1 | 0,004 |  |
| Chicken |  |  | Galinha (Port.) | m |  |  | 1 | 0,004 |  |
| Eggs |  |  | Ovos (Port.) |  |  |  | 1 | 0,004 | Child becomes sick |
| Fish |  |  | Peixe (Port.) |  | Dried | 37 | 38 | 0,161 | Milk becomes watery and child suffers from diarrhoea |
|  |  |  |  |  | n | 1 |  |  |  |
| Goat |  |  | Cabra (Port.) | m |  |  | 10 | 0,042 | Child becomes scrabies; child dies |
| Meat with vegetables |  |  |  |  |  |  | 1 | 0,004 |  |
| Pork |  |  | Porco (Port.) | m |  |  | 1 | 0,004 |  |
| Salt |  |  | Sal (Port.) |  |  |  | 1 | 0,004 |  |
|  |  |  | Casca (Port.) |  |  |  | 1 | 0,004 | Don't use during pregnancy |
|  |  |  | Mabele (Kik.) | n |  |  | 1 | 0,004 |  |
|  |  |  | Nganzi (Kik.) | n |  |  | 1 | 0,004 | Don't use during pregnancy |

Table 8: Overview of plants, foods and treatments, which are used to decrease the breast milk production. All identified plant species were categorised in endemic (E), naturalised (*), listed (+) and not listed (-) (Neuwinger 2000). For most plants, the herbarium number according to Herbarium Dresdense (DR) is listed. In cases where no herbarium specimen was collected the number of a photo proof (F or G-F) is given. Plants which were identified with the comparison of the Portuguese or Kikongo names with the data collection of Lautenschläger (2018)19 were marked with an •; part: b = bark, ba = bacon, bo = bone, cl = clay, f = fruit, l = leaves, o = oil, p = whole plant, r = root, s= seed, ss = stem sap, st = stem; n = no further information; Total number of citations in the interviews (CI), number of interviews (N), KiKongo name (Kik), Portuguese name (Port.)

| **Scientific name** | **Herbarium Number DR** | **Family** | **Local name** | **Part** | **Preparation** | **Citations** | **CI** | **RFC** (N = 124) |
| --- | --- | --- | --- | --- | --- | --- | --- | --- |
| *^+^Abrus precatorius* L. | 051649 | Fabaceae | Kiambiembie (Kik.) | l | Maceration | 1 | 2 | 0,016 |
|  |  |  |  | n |  | 1 |  |  |
| *^-^Canavalia gladiata* (Jacq.) DC. | F_19 | Fabaceae | Nkasa (Kik.), Feijão (Port.) | s | Maceration with Ntowani (*Phyllanthus* spec.) leaves and Ngidingonda(a specific stone) |  | 1 | 0,008 |
| *Costus* spec. | 051608 | Costaceae | Nsangalavua (Kik.) | n |  |  | 1 | 0,008 |
| *^+^Crassocephalum rubens* (Juss. ex Jacq.) S.Moore | 051645 | Asteraceae | Bungudia (Kik.) | n |  |  | 1 | 0,008 |
| *Cucurbita* spec. |  | Cucurbitaceae | Muteta, Abóbora (Port.), Muengeleka (Kik.) | n |  |  | 1 | 0,008 |
| *^+^Elaeis guineensis* Jacq. | G-F3  G-F4  G-F5 | Arecaceae | Kokonote (Kik.) | s | With Mandioca (*Manihot esculenta*) and cana de açúcar (*Saccharum officinarum*) |  | 1 | 0,008 |
| *^+^Leonotis nepetifolia* (L.) R.Br. | 051606 | Lamiaceae | Manuansongi (Kik.) | l | Maceration |  | 1 | 0,008 |
| *^-^Manihot esculenta* Crantz | 042760 | Euphorbiaceae | Mandioca (Port.) | r | With Kokonote (*Elaeis guineensis*) and Cana de açúcar (*Saccharum officinarum*) |  | 1 | 0,008 |
| *^+^Maprounea africana* Müll.Arg. | 051640 | Euphorbiaceae | Nsele nsiele (Kik.) | l | Maceration |  | 1 | 0,008 |
| *^+^Milicia excelsa* (Welw.) C.C.Berg | 051637 | Moraceae | Nkamba (Kik.), Moreira (Port.) | ss |  |  | 1 | 0,008 |
| *^+^Monodora myristica* (Gaertn.) Dunal | 044707 | Annonaceae | Mpeve (Kik.) | s | Masticate raw and apply on the breast | 1 | 3 | 0,024 |
|  |  |  |  | n | Masticate raw | 1 |  |  |
|  |  |  |  |  | n | 1 |  |  |
| *Phyllanthus* spec. | 051651 | Phyllanthaceae | Ntowani (Kik.) | l | Maceration with Nkasa (*Canavalia gladiata*) seeds and Ngidingonda (a specific stone) |  | 1 | 0,008 |
| *^*^Saccharum officinarum* L*.* |  | Poaceae | Cana de açúcar (Port.) | st | With Kokonote (*Elaeis guineensis*) and Mandioca (*Manihot esculenta*) | 1 | 2 | 0,016 |
|  |  |  |  |  | n | 1 |  |  |
| ^+^*Salacia erecta* (G.Don) Walp. | 053479  053480 | Celastraceae | Mbonda (Kik.) | l | Infusion | 1 | 2 | 0,016 |
|  |  |  |  |  | n | 1 |  |  |
| *^+^Sesamum indicum L.* | 043895 | Pedaliaceae | Gergelim (Port.), Wanguila (Kik.) | S |  |  | 1 | 0,008 |
| *^+^Vernonia amygdalina* Delile | 051619 | Asteraceae | Malulu, Malulua (Kik.) | l | Infusion |  | 1 | 0,008 |
| *^+^Xylopia aethiopica* (Dunal) A.Rich. | F_71  044264 | Annonaceae | Nkuwa nkuwa, Nkuakua (Kik.) | n |  |  | 1 | 0,008 |
| Clean the body |  |  |  |  |  |  | 2 | 0,016 |
| Express breast milk |  |  |  |  |  |  | 1 | 0,008 |
| Fish |  |  | Peixe (Port.) |  | Dried |  | 1 | 0,008 |
| massage |  |  |  |  |  |  | 1 | 0,008 |
| Reduced food intake |  |  |  |  |  |  | 2 | 0,016 |
|  |  |  | Bulukutu (Kik.) |  | Infusion |  | 1 | 0,008 |
|  |  |  | Kangudi (Kik.) |  |  |  | 1 | 0,008 |
|  |  |  | Ngidingonda (Kik.) | A specific kind of stone | Maceration with Ntowani (*Phyllanthus* spec.) leaves and Nkasa (*Canavalia gladiata*) seeds |  | 1 | 0,008 |
